# Prevalence and Correlates of Caregiver-Reported Disordered Eating Behaviors and Concerns Among US Children and Adolescents Aged 6 to 17 Years, 2022

**DOI:** 10.5888/pcd23.250353

**Published:** 2026-06-18

**Authors:** Lydie A. Lebrun-Harris, Ariel B. Beccia, S. Bryn Austin, Jason M. Fields

**Affiliations:** 1 US Department of Health and Human Services, Health Resources and Services Administration, Maternal and Child Health Bureau, Rockville, Maryland; 2 Boston Children’s Hospital, Boston, Massachusetts; 3 Harvard Medical School, Boston, Massachusetts; 4 Harvard T.H. Chan School of Public Health, Boston, Massachusetts; 5 US Census Bureau, Social, Economic, and Housing Statistics Division, Washington, District of Columbia

## Abstract

**Introduction:**

Disordered eating behaviors are increasingly recognized among children and adolescents in the US, making it critical to understand their prevalence and associated risk factors to support early identification and intervention. The objective of this study was to estimate the prevalence and correlates of caregiver-reported disordered eating behaviors and concerns among US children and adolescents aged 6 to 17 years.

**Methods:**

We analyzed data from the 2022 National Survey of Children’s Health (n = 34,362), estimating age- and sex-stratified prevalence of past-year child’s concerns about body weight, shape, or size; disordered eating behaviors; and caregiver concerns about those behaviors. We conducted bivariate analyses identifying sociodemographic, economic, health-related, and caregiver/family-related correlates.

**Results:**

The most prevalent behaviors were extremely picky eating (24.5% among children aged 6–11 y; 19.3% among adolescents aged 12–17 y), low interest in food (11.1% and 10.6%, respectively), and skipping meals/fasting (6.8% and 13.4%, respectively). About one-quarter of caregivers were “very much” or “somewhat” concerned about their child’s behaviors. Several factors were associated with disordered eating behaviors/concerns, including food insufficiency; mental/emotional/behavioral conditions; frequent bullying; high levels of screentime; worse caregiver mental/emotional health; and adverse childhood experiences.

**Conclusion:**

Based on caregiver reports, nearly one-third of children and adolescents in our study population engaged in at least 1 form of disordered eating in the past year. Caregivers may be well-positioned to observe and report early behavior changes, potentially enabling earlier clinical assessment and intervention and improved prognosis.

**Summary**

**What is already known about this topic?**

Up-to-date national estimates of eating disorders and disordered eating among US children and adolescents are lacking, especially for children younger than 12 years.

**What is added by this report?**

This study provides the first nationally representative estimates of parent- or caregiver-reported observations and concerns regarding disordered eating among children and adolescents aged 6 to 17 years. Disordered eating behaviors affected almost one-third of the study population. Caregivers expressed concern in about one-quarter of cases. Several social and health-related factors were significantly associated with these behaviors and concerns.

**What are the implications for public health practice?**

Results suggest a need for ongoing population-based data collection; prevention, screening, diagnosis, and treatment; and increased awareness and education among caregivers and health care providers.

## Introduction

Only a few national data sources are available to provide up-to-date epidemiologic information on eating disorders among children and adolescents, despite growing public health concerns and substantial health consequences and economic costs (1,2). From 2001 through 2004, the estimated lifetime prevalence of eating disorders (anorexia nervosa, bulimia nervosa, binge eating) among US adolescents aged 13 to 18 years was 2.7% (3). A more recent analysis that included the diagnosis of other specified feeding and eating disorders, based on nationally representative surveys from 2007 and 2011, indicated a much higher prevalence of eating disorders, peaking at age 21 years (10.3% among females, 7.4% among males) (4).

Subthreshold disordered eating behaviors are also highly prevalent among young people (5). A meta-analysis of 32 studies across 16 countries published from 1999 through 2022 estimated that 22% of young people aged 6 to 18 years screened positive for disordered eating (6). In the US, the biennial Youth Risk Behavior Survey (YRBS) previously assessed past-month fasting, purging, and diet pill use, but those questions were removed from the questionnaire in 2015 (7). The most recent YRBS estimates, from 2013, showed a prevalence of disordered eating among high school students ranging from 20% to 29% among girls and 8% to 13% among boys, depending on race and ethnicity (8). Other research also indicates that eating disorder and disordered eating caseloads in hospitals and emergency departments doubled during the COVID-19 pandemic (9–11), underscoring the need for surveillance data.

A gap in knowledge pertaining to young children also exists, despite recent findings that prodromal symptoms (eg, concerns over body weight, shape, or size) may emerge as early as 6 years of age (12). Moreover, evidence is growing of a disproportionate prevalence among girls, young people belonging to racial and ethnic minority groups, and young people experiencing food insecurity and other forms of socioeconomic disadvantage (8,13,14). However, nationally representative estimates of the current prevalence among subgroups of US children are lacking.

While most data on disordered eating behaviors among young people are obtained through self-report, data reported by parents or caregivers (henceforth “caregivers”) are also informative, particularly in contexts where young people may deny, minimize, or fail to recognize symptoms. Young people with eating disorders may underreport symptoms and behaviors compared with their caregivers (15,16). However, caregivers may be better positioned to observe and report early changes in certain behaviors, especially for younger children, which can potentially lead to professional assessments, earlier intervention, and improved prognosis (17). In addition, caregiver perspectives may offer a broader context of the child’s environment (eg, family stressors, resources), which may influence disordered eating.

The Health Resources and Services Administration’s (HRSA’s) Maternal and Child Health Bureau added content on the National Survey of Children’s Health (NSCH) in 2022 to capture caregiver-reported disordered eating behaviors and concerns among children and adolescents aged 6 to 17 years. This study sought to answer the following questions: 1) What is the prevalence of concerns among children and adolescents about their body weight, shape, or size, as reported by their caregivers? 2) What is the prevalence of caregiver-reported disordered eating behaviors among children and adolescents? 3) What is the prevalence of caregiver concerns about their child’s disordered eating behaviors? and 4) How does the prevalence of caregiver-reported disordered eating behaviors and concerns differ by sociodemographic, economic, health-related, and family characteristics?

## Methods

### Data source

We analyzed cross-sectional data from the NSCH, an annual, address-based probability survey of noninstitutionalized children from birth through 17 years in all 50 US states and the District of Columbia, funded and directed by the Maternal and Child Health Bureau and administered by the US Census Bureau. The 2022 NSCH drew a stratified random sample of approximately 360,000 residential addresses from the Census Bureau’s Master Address File, using administrative-record flags to oversample addresses likely to include children. During data collection, a household screener identified eligible children, and 1 child was randomly selected in each household to be the subject of an age-specific topical questionnaire completed by a parent or caregiver via web or paper, with telephone assistance available. Child-level survey weights, constructed by the US Census Bureau, incorporate the probability of selection, nonresponse adjustments, and poststratification (raking) to state-level and national-level population controls, allowing estimates to be generalized to the US population of noninstitutionalized children. Detailed survey procedures are described elsewhere (18). Data are publicly available and deidentified; therefore, institutional review board approval for human subjects research did not apply. The interview completion rate (ie, the probability that an occupied household who initiated the survey completed both the screener and topical questionnaires) was 79%. The overall response rate (ie, the probability that a sampled address was confirmed to be an occupied household and completed the survey) was 39% (19). The analytic sample included 15,334 children aged 6 to 11 years and 19,028 adolescents aged 12 to 17 years. Records with missing data on disordered eating behaviors and concerns were excluded from analyses; missingness ranged from 0.2% (caregiver concerns about disordered eating behaviors) to 1.2% (binge eating).

### Measures

We examined caregiver reports of their child’s body-related concerns by using the question, “During the past 12 months, how concerned was this child about their weight, body shape, or body size?” and identified the proportion of respondents who answered, “very much,” “somewhat,” or “not at all.”

Caregivers reported whether their child engaged in any of 8 disordered eating behaviors during the past 12 months: 1) skipping meals or fasting (excluding for religious reasons); 2) purging or vomiting after eating; 3) using diet pills, laxatives, or diuretics to lose or maintain weight without a doctor’s orders; 4) low interest in food; 5) extremely picky eating; 6) not eating due to fear of vomiting or choking; 7) binge eating; and 8) overexercising. We created a combined “restrictive eating” category that included skipping meals or fasting, purging or vomiting and using diet pills, laxatives, or diuretics, and a combined “avoidant eating” category that included low interest in food, extremely picky eating, and not eating due to vomiting/choking fears.

Caregivers who reported their child engaged in at least 1 disordered eating behavior were then asked, “During the past 12 months, how concerned were you about this child engaging in these behaviors?” We identified the proportion of respondents who answered, “very much,” “somewhat,” or “not at all.”

Correlates included sociodemographic, economic, health-related, and family factors. Sociodemographic characteristics included child sex, race and ethnicity, and household language. Economic factors included family income-to-poverty ratio as a percentage of the federal poverty level (FPL), food insufficiency, and health insurance status. Health-related factors included child’s general health status; sex-specific body mass index (BMI) percentile categories; current mental, emotional, or behavioral conditions (depression, anxiety, behavioral or conduct problems, autism spectrum disorder, attention deficit/hyperactivity disorder [ADHD]); bullying victimization; screentime during weekdays (excluding schoolwork); usual source of sick care; personal doctor or nurse; preventive medical visit in the past year; and whether a doctor ever told the caregiver their child was overweight. Family characteristics included highest education level of caregivers in the household, caregiver mental and emotional health, caregiver concerns about their child’s weight, how well caregiver and child share ideas and talk together, frequency of family meals, and number of adverse childhood experiences.

### Statistical analysis

We estimated prevalence and 95% CIs for the measures of caregiver-reported disordered eating behaviors and concerns among children aged 6 to 11 years and adolescents aged 12 to 17 years, overall and by sex for each age group. To account for complex sample design, all estimates were based on a multistage weighting process and iterative raking to population controls from American Community Survey estimates. Within each age group, we calculated the observed prevalence among subpopulations and conducted Rao–Scott design-adjusted χ^2^ tests of independence to assess the associations between sociodemographic, economic, health-related, and family factors and each measure. Because we found sex differences in the prevalence estimates among adolescents, we further stratified the bivariate analyses by sex for this age group.

We conducted analyses using Stata/SE version 18.0 (StataCorp LLC). Statistical significance was assessed by using a 2-sided test (α =.05) and 95% CIs, with no adjustments made for multiple comparisons due to the descriptive nature of the analysis. The public use file provided imputed data for missing values for sex (0.1% missing), ethnicity (0.3% missing), race (1.9% missing), and family income (19.8% missing).

## Results

### Prevalence of disordered eating behaviors

The study sample (unweighted n = 34,362) was 51.2% male and 48.8% female; 48.3% were aged 6 to 11 years and 51.7% were aged 12 to 17 years; 47.7% were non-Hispanic White, 13.1% non-Hispanic Black, and 26.8% Hispanic of any race (Table 1). About one-third of children (31.1%) (Table 2A) and adolescents (30.4%) (Table 2B) were reported by their caregiver as displaying 1 or more disordered eating behaviors, representing about 15.4 million US children and adolescents. The prevalence of any disordered eating behavior was significantly higher among adolescent females than males (33.1%; 95% CI, 31.2%–35.0% vs 27.7%; 95% CI, 26.1%–29.6%, *P* < .001).

**Table 1:** Characteristics of US Children and Adolescents Aged 6 to 17 Years, National Survey of Children’s Health, 2022

Characteristic	Unweighted no.	Weighted frequency	Weighted % (95% CI)
**Total**	34,362	50,725,918	100.0
**Sociodemographic and economic characteristics**			
**Age, y**			
6–11	15,334	24,482,676	48.3 (47.2–49.3)
12–17	19,028	26,243,242	51.7 (50.7–52.8)
**Sex**			
Male	17,830	25,975,093	51.2 (50.2–52.3)
Female	16,532	24,750,825	48.8 (47.7–49.8)
**Race and ethnicity**			
Hispanic, Latino, or Spanish origin, any race	5,356	13,607,155	26.8 (25.8–27.9)
Non-Hispanic American Indian/Alaska Native	205	310,688	0.6 (0.5–0.8)
Non-Hispanic Asian	2,139	2,540,371	5.0 (4.6–5.4)
Non-Hispanic Black	2,291	6,621,278	13.1 (12.3–13.9)
Non-Hispanic Native Hawaiian and Other Pacific Islander	93	90,767	0.2 (0.1–0.3)
Non-Hispanic White	21,944	24,201,507	47.7 (46.7–48.7)
Non-Hispanic multiple races	2,334	3,354,152	6.6 (6.2–7.1)
**Household language**			
English	31,290	42,741,365	85.1 (84.1–86.0)
Spanish	1,579	5,170,406	10.3 (9.4–11.2)
Other	1,279	2,327,438	4.6 (4.1–5.2)
**Family income-to-poverty ratio, % federal poverty level**			
<100	4,520	9,282,716	18.5 (17.3–19.7)
100–199	5,657	10,061,203	19.9 (18.8–21.0)
200–399	9,964	14,688,490	28.9 (27.9–29.9)
≥400	14,221	16,693,510	32.7 (31.8–33.7)
**Food insufficiency, past year**			
Always could afford nutritious meals	23,755	32,473,040	66.0 (65.0–67.1)
Always could afford enough to eat, but not always nutritious food	8,473	13,990,416	28.4 (27.4–29.5)
Often or sometimes could not afford enough to eat	1,285	2,730,405	5.6 (5.0–6.1)
**Health insurance status**			
Private only	22,712	28,745,986	58.0 (56.9–59.1)
Public only or public with private	9,492	17,270,821	34.9 (33.8–35.9)
Uninsured	1,479	3,511,694	7.1 (6.4–7.9)
**Health status, health-related behaviors, and health care characteristics**			
**General health status**			
Excellent/very good	30,780	44,763,477	88.5 (87.7–89.2)
Good/fair/poor	3,492	5,825,585	11.5 (10.8–12.3)
**Body mass index**			
Less than 5th percentile	2,831	4,416,182	9.3 (8.6–10.0)
5th to less than 85th percentile	20,293	27,677,821	58.1 (57.0–59.1)
85th to less than 95th percentile	4,783	7,142,440	15.0 (14.2–15.8)
95th or greater percentile	4,992	8,444,580	17.7 (16.9–18.6)
**Current mental, emotional, or behavioral conditions^a^**			
Depression	2,478	2,810,309	5.6 (5.2–6.0)
Anxiety problems	5,428	6,291,019	12.5 (11.9–13.2)
Behavioral or conduct problems	3,005	4,060,463	8.0 (7.5–8.6)
Autism spectrum disorder	1,355	1,750,953	3.5 (3.1–3.9)
ADHD	4,847	6,198,700	12.4 (11.7–13.0)
**Bullying victimization, past year**			
Never	19,483	31,059,116	62.5 (61.5–63.5)
1–2 times in the past year	9,758	13,159,841	26.5 (25.6–27.4)
1–2 times per month	2,583	2,987,656	6.0 (5.6–6.5)
1–2 times per week	1,331	1,706,986	3.4 (3.1–3.8)
Almost every day	661	789,866	1.6 (1.4–1.8)
**Screentime during weekdays (excluding schoolwork), no. of hours**			
≤1	6,656	9,882,793	19.8 (19.0–20.7)
2–3	17,722	25,739,024	51.7 (50.6–52.7)
≥4	9,436	14,181,516	28.5 (27.5–29.5)
**Health care factors**			
Usual source of sick care	25,600	34,867,181	73.8 (72.7–74.8)
Personal doctor or nurse	25,826	35,530,454	70.6 (69.5–71.6)
≥1 Preventive medical visit, past year	21,476	29,585,681	73.2 (72.1–74.4)
Doctor ever told caregiver their child is overweight	2,955	4,942,592	9.8 (9.2–10.5)
**Family characteristics**			
**Highest household education**			
Less than high school diploma	1,042	4,696,630	9.3 (8.4–10.2)
High school diploma	4,794	9,635,738	19.0 (18.1–19.9)
Some college	7,627	10,461,204	20.6 (19.8–21.5)
College degree or higher	20,899	25,932,346	51.1 (50.1–52.2)
**Caregiver mental and emotional health**			
One or both adults excellent/very good	19,996	29,961,734	62.0 (61.0–63.0)
At least 1 adult good/fair/poor	13,030	18,368,612	38.0 (37.0–39.1)
**Caregiver concern about child’s weight**			
Yes, concerned it’s too high	2,993	4,679,833	9.3 (8.7–9.9)
Yes, concerned it’s too low	1,134	1,645,004	3.3 (2.9–3.7)
No, not concerned	30,058	44,087,919	87.5 (86.7–88.2)
**Caregiver and child share ideas or talk**			
Very well	20,387	30,541,660	61.6 (60.6–62.7)
Somewhat well	11,553	16,374,736	33.1 (32.1–34.1)
Not very well or not well at all	1,765	2,635,940	5.3 (4.8–5.8)
**Family eats meals together, days per week**			
0	1,492	2,145,464	4.3 (3.9–4.8)
1–3	9,099	13,347,987	26.9 (26.0–27.9)
4–6	10,971	14,724,022	29.7 (28.8–30.6)
7	12,126	19,381,728	39.1 (38.0–40.1)
**Number of ACEs^b^**			
0	20,975	30,711,636	60.5 (59.5–61.6)
1	7,207	11,121,734	21.9 (21.0–22.8)
≥2	6,180	8,892,548	17.5 (16.8–18.3)

Abbreviations: ACE, adverse childhood experience; ADHD, attention deficit/hyperactivity disorder.

a Percentages may add up to more than 100% due to comorbid conditions.

b Of 9 possible ACEs: parents divorced or separated; parent died; parent incarcerated; interpersonal violence in the home; victim or witness of neighborhood violence; lived with someone who was mentally ill, severely depressed, or suicidal; lived with someone with an alcohol or drug problem; treated unfairly because of race or ethnicity; treated unfairly because of disability or health condition.

**Table 2A:** Caregiver-Reported Prevalence of Disordered Eating–Related Behaviors and Concerns Among US Children Aged 6 to 11 Years, by Age and Sex, National Survey of Children’s Health, 2022

Behavior or concern	All (N = 15,334)		Male, weighted % (95% CI) (n = 7,952)	Female, weighted % (95% CI) (n = 7,382)	Male vs female, *P* value^a^
	Unweighted no.	Weighted frequency (weighted %) [95% CI]			
**Child has concerns about their weight, body shape, or body size, past year**					
Very much	235	436,266 (1.8) [1.4–2.3]	1.4 (1.0–1.8)	2.3 (1.6–3.2)	.04
Somewhat	2,183	3,345,235 (13.8) [12.9–14.9]	13.2 (11.9–14.5)	14.5 (13.1–16.1)	
Not at all	12,798	20,405,511 (84.4) [83.3–85.4]	85.5 (84.1–86.8)	83.2 (81.5–84.8)	
**Disordered eating behaviors, past year**					
Any (≥1) disordered eating behavior	4,916	7,559,758 (31.1) [29.7–32.5]	31.2 (29.3–33.1)	30.9 (28.8–33.0)	.83
Restrictive eating behaviors					
Skipping meals or fasting	1,093	1,647,319 (6.8) [6.1–7.5]	7.4 (6.4–8.4)	6.2 (5.3–7.2)	.09
Purging or vomiting after eating	88	151,969 (0.6) [0.4–0.9]	0.7 (0.4–1.0)	0.6 (0.3–1.1)^b^	.70
Using diet pills, laxatives, or diuretics	12^b^	19,441 (0.1) [0.03–0.2]^b^	—c	—c	—c
Avoidant eating behaviors					
Low interest in food	1,774	2,681,735 (11.1) [10.1–12.1]	11.1 (9.9–12.4)	11.0 (9.5–12.8)	.96
Extremely picky eating	3,974	5,945,836 (24.5) [23.2–25.9]	24.9 (23.2–26.7)	24.1 (22.3–26.1)	.57
Not eating due to fear of vomiting or choking	147	178,026 (0.7) [0.6–1.0]	0.7 (0.5–1.1)	0.8 (0.6–1.1)	.65
Binge eating	540	915,052 (3.8) [3.3–4.4]	3.7 (3.1–4.2)	3.9 (3.1–4.8)	.81
Overexercising	54	122,875 (0.5) [0.3–0.8]	0.7 (0.4–1.1)	0.4 (0.2–0.7)^b^	.17
**Caregiver concerns about child’s disordered eating behaviors, past year^d^**					
Very much	218	386,335 (5.2) [4.0–6.6]	4.7 (3.3–6.7)	5.6 (4.0–7.8)	.20
Somewhat	1,101	1,660,602 (22.1) [20.1–24.3]	24.0 (21.2–27.1)	20.1 (17.3–23.3)	
Not at all	3,567	5,460,104 (72.7) [70.3–75.0]	71.3 (68.0–74.3)	74.3 (70.8–77.6)	

a Determined by Rao–Scott design-adjusted χ^2^ test of independence at significance level of .05.

b Interpret estimate with caution (relative SE >30%).

c Estimate suppressed due to unreliability (relative SE >50%).

d Among children whose caregiver reported ≥1 of the 8 listed disordered eating behaviors.

**Table 2B:** Caregiver-Reported Prevalence of Disordered Eating–Related Behaviors and Concerns Among US Adolescents Aged 12 to 17 Years, by Age and Sex, National Survey of Children’s Health, 2022

Concern or behavior	All (N = 19,028)		Male, weighted % (95% CI) (n = 9,878)	Female, weighted % (95% CI) (n = 9,150)	Male vs female, *P* value^a^
	Unweighted no.	Weighted frequency (weighted %) [95% CI]			
**Child has concerns about their weight, body shape, or body size, past year**					
Very much	1,135	1,449,775 (5.6) [5.0–6.3]	5.0 (4.1–6.0)	6.3 (5.5–7.2)	.03
Somewhat	5,519	7,165,370 (27.7) [26.4–29.0]	26.8 (24.9–28.6)	28.6 (26.9–30.4)	
Not at all	12,177	17,292,875 (66.8) [65.4–68.1]	68.3 (66.3–70.2)	65.1 (63.2–67.0)	
**Disordered eating behaviors, past year**					
Any (≥1) disordered eating behavior	5,994	7,919,850 (30.4) [29.1–31.7]	27.7 (26.1–29.6)	33.1 (31.2–35.0)	<.001
Restrictive eating behaviors					
Skipping meals or fasting	2,810	3,474,890 (13.4) [12.5–14.3]	11.1 (10.0–12.3)	15.7 (14.5–17.1)	<.001
Purging or vomiting after eating	126	150,973 (0.6) [0.4–0.8]	0.4 (0.3–0.7)	0.7 (0.5–1.0)	.07
Using diet pills, laxatives, or diuretics	54	48,207 (0.2) [0.1–0.3]	0.1 (0.1–0.3)^b^	0.2 (0.2–0.5)^b^	.26
Avoidant eating behaviors					
Low interest in food	2,256	2,754,528 (10.6) [9.8–11.4]	8.0 (7.1–9.0)	13.3 (12.1–14.7)	<.001
Extremely picky eating	3,837	5,013,261 (19.3) [18.2–20.4]	17.2 (15.8–18.7)	21.5 (19.8–23.2)	.001
Not eating due to fear of vomiting or choking	181	184,918 (0.7) [0.6–0.9]	0.4 (0.2–0.6)	1.1 (0.8–1.5)	<.001
Binge eating	1,060	1,522,267 (5.9) [5.2–6.7]	5.8 (4.8–7.0)	6.0 (5.1–7.0)	.83
Overexercising	315	411,068 (1.6) [1.3–2.0]	1.7 (1.3–2.3)	1.5 (1.1–2.0)	.44
**Caregiver concerns about child’s disordered eating behaviors, past year^c^**					
Very much	355	401,600 (5.1) [4.3–6.1]	4.5 (3.3–6.0)	5.7 (4.6–7.0)	.01
Somewhat	1,594	2,048,503 (26.1) [24.0–28.4]	23.3 (20.3–26.6)	28.6 (25.6–31.8)	
Not at all	4,002	5,390,544 (68.8) [66.4–71.0]	72.2 (68.8–75.4)	65.7 (62.5–68.9)	

a Determined by Rao–Scott design-adjusted χ^2^ test of independence at significance level of .05.

b Interpret estimate with caution (relative SE >30%).

c Among children whose caregiver reported ≥1 of the 8 listed disordered eating behaviors.

Among children aged 6 to 11 years, caregivers reported that 1.8% were “very much” and 13.8% were “somewhat” concerned about their body weight, shape, or size (Table 2A). The most prevalent disordered eating behaviors observed were extremely picky eating (24.5%) and low interest in food (11.1%). Among children engaged in 1 or more behaviors in the past year, 5.2% of caregivers reported being “very much” concerned about their child’s behaviors and another 22.1% were “somewhat” concerned.

Among adolescents aged 12 to 17 years, caregivers reported that 5.6% were “very much” and 27.7% were “somewhat” concerned about their body weight, shape, or size (Table 2B). The most prevalent disordered eating behaviors observed were extremely picky eating (19.3%), skipping meals or fasting (13.4%), and low interest in food (10.6%). Among adolescents engaged in 1 or more behaviors in the past year, 5.1% of caregivers reported being “very much” concerned about their adolescent’s behaviors and another 26.1% were “somewhat” concerned.

The prevalence of caregiver-reported avoidant eating behaviors (ie, low interest in food, extremely picky eating, not eating due to fear of vomiting or choking) was higher among children than adolescents, while skipping meals or fasting, binge eating, and overexercising were higher among adolescents than children. The prevalence of concerns about their body weight, shape, or size was also higher among adolescents than children; however, the prevalence of caregiver concerns about behaviors was similar for both age groups. We found more sex differences among adolescents than among children: compared with adolescent males, adolescent females were reported to have a higher prevalence of skipping meals or fasting, low interest in food, extremely picky eating, and not eating due to fear of vomiting or choking.

### Correlates of disordered eating behaviors among children

Among children aged 6 to 11 years, we observed a higher prevalence of most caregiver-reported disordered eating behaviors and related concerns among those experiencing food insufficiency and having public health insurance, compared with food secure and privately insured children, respectively (Table 3A). Non-Hispanic Asian children had lower prevalence of avoidant eating behaviors (19.9%; 95% CI, 15.9%–24.7%) than non-Hispanic White children (26.6%; 95% CI, 25.0%–28.1%). Caregivers of children from lower-income households were also more likely to report that their child binge ate (<100% FPL: 5.4%; 95% CI, 3.8%–7.1%), compared with caregivers of children from higher income households (≥ 400% FPL: 2.4%; 95% CI, 1.5%–3.3%) (Table 3B). Children in good/fair/poor health; children with current mental, emotional, or behavioral conditions; children experiencing more frequent bullying victimization; children with higher levels of screentime; and children whose doctor ever told their caregivers that they were overweight were also found to have a higher prevalence of most outcomes compared with their counterparts who did not experience these factors. Compared with children with lower BMI percentile, those with higher BMI percentile were more likely to have caregivers report that their child binge ate and that their child had concerns about their body weight, shape, or size. In terms of family factors, children whose caregivers had lower levels of education, children with caregivers in good/fair/poor mental/emotional health, children whose caregivers had concerns about their child’s weight, children whose caregivers did not share ideas or talk together, children from families that never or rarely eat meals together, and children with 1 or more adverse childhood experiences were observed to have a higher prevalence of most caregiver-reported outcomes compared with their counterparts.

**Table 3A:** Caregiver-Reported Prevalence of Disordered Eating–Related Behaviors and Concerns Among Children Aged 6 to 11 Years, by Sociodemographic, Economic, Health-Related, and Family Characteristics, National Survey of Children’s Health, 2022^a^

Characteristic	Child “very much” concerned about their weight, body shape, or body size, past year		Any restrictive eating behaviors, past year^b^		Any avoidant eating behaviors, past year^c^	
	Unweighted no. (weighted %) [95% CI]	*P* d	Unweighted no. (weighted %) [95% CI]	*P* d	Unweighted no. (weighted %) [95% CI]	*P* d
Total	235 (1.8) [1.4–2.3]	NA	1,164 (7.3) [6.6–8.1]	NA	4,492 (27.9) [26.5–29.3]	NA
**Sociodemographic and economic characteristics**						
**Race and ethnicity**						
Hispanic, Latino, or Spanish origin, any race	52 (2.3) [1.5–3.7]	.001	205 (8.5) [6.8–10.6]	.02	707 (30.6) [27.1–34.3]	.01
Non-Hispanic American Indian/Alaska Native	—e		—e		32 (30.9) [19.7–45.0]	
Non-Hispanic Asian	18 (1.9) [0.9–3.9]^f^		57 (6.7) [4.4–10.1]		208 (19.9) [15.9–24.7]	
Non-Hispanic Black	27 (3.9) [2.1–7.4]^f^		84 (9.6) [7.2–12.6]		305 (28.9) [24.8–33.5]	
Non-Hispanic Native Hawaiian and Other Pacific Islander	—e		—e		11 (28.9) [15.0–48.5]^f^	
Non-Hispanic White	116 (1.0) [0.8–1.4]		736 (6.5) [5.7–7.3]		2,869 (26.6) [25.0–28.1]	
Non-Hispanic multiple races	21 (1.9) [1.1–3.1]		76 (5.5) [4.0–7.5]		360 (30.8) [26.6–35.3]	
**Household language**						
English	196 (1.8) [1.4–2.4]	.51	1,076 (7.5) [6.7–8.3]	.39	4,156 (28.6) [27.2–30.0]	.10
Spanish	20 (1.3) [0.7–2.2]		37 (5.9) [3.7–9.2]		159 (26.1) [20.4–32.7]	
Other	17 (2.3) [1.0–5.1]^f^		42 (6.0) [3.8–9.5]		150 (20.7) [15.8–26.7]	
**Family income-to-poverty ratio, % of federal poverty level**						
<100	59 (3.3) [1.3–5.2]	.16	172 (7.9) [5.8–9.9]	.73	656 (29.7) [25.5–33.8]	.21
100–199	48 (1.8) [0.4–3.3]^f^		979 (7.9) [5.9–10.0]		787 (27.9) [24.6–30.2]	
200–399	70 (1.5) [0.7–2.3]		353 (6.8) [5.5–8.0]		1,331 (28.9) [26.2–31.6]	
≥400	58 (1.3) [0.7–1.8]		414 (7.1) [5.9–8.3]		1,718 (25.9) [24.1–27.8]	
**Food insufficiency, past year**						
Always could afford nutritious meals	113 (1.7) [1.2–2.4]	.02	638 (5.5) [4.8–6.3]	<.001	2,722 (23.8) [22.3–25.5]	<.001
Always could afford enough to eat, but not always nutritious food	92 (1.7) [1.3–2.4]		409 (10.6) [8.9–12.5]		1,426 (35.9) [33.1–38.8]	
Often or sometimes could not afford enough to eat	23 (4.0) [2.3–7.0]		98 (14.3) [10.2–19.6]		329 (40.5) [32.9–48.6]	
**Health insurance status**						
Private only	112 (1.0) [0.8–1.4]	<.001	684 (6.8) [6.0–7.7]	<.001	2,736 (26.0) [24.5–27.5]	.04
Public (alone or with private)	110 (3.2) [2.3–4.5]		439 (8.9) [7.6–10.5]		1,542 (31.3) [28.8–33.9]	
Uninsured	7 (0.3) [0.1–0.7]^f^		31 (3.7) [2.2–6.2]		156 (27.1) [19.2–36.8]	
**Health status, health-related behaviors, and health care characteristics**						
**General health status**						
Excellent/very good	144 (1.3) [0.9–1.8]	<.001	942 (6.3) [5.7–7.1]	<.001	3,863 (26.1) [24.7–27.6]	<.001
Good/fair/poor	89 (6.6) [4.7–9.1]		219 (16.6) [13.4–20.5]		615 (44.9) [39.7–50.3]	
**Body mass index**						
Less than 5th percentile	11 (0.6) [0.3–1.2]^f^	<.001	151 (8.8) [6.6–11.6]	.33	591 (32.3) [28.4–36.4]	.07
5th to less than 85th percentile	55 (0.7) [0.5–1.0]		612 (6.9) [6.00–7.9]		2,325 (27.6) [25.7–29.5]	
85th to less than 95th percentile	39 (2.1) [1.3–3.5]		144 (6.4) [4.9–8.3]		572 (24.9) [21.4–28.9]	
95th or greater percentile	109 (4.7) [3.3–6.5]		183 (7.6) [5.9–9.7]		710 (27.5) [24.4–30.9]	
**Current mental, emotional, or behavioral conditions**						
**Depression**						
Yes	36 (11.3) [6.7–18.2]	<.001	107 (24.4) [18.3–31.8]	<.001	580 (55.7) [45.9–65.1]	<.001
No	195 (1.6) [1.2–2.1]		1,047 (6.8) [6.2–7.6]		4,235 (27.3) [25.9–28.7]	
**Anxiety problems**						
Yes	76 (4.6) [3.2–6.4]	<.001	323 (19.0) [15.7–22.9]	<.001	918 (54.4) [49.3–59.3]	<.001
No	152 (1.5) [1.1–2.1]		826 (6.1) [5.5–6.9]		3,532 (25.3) [23.9–26.7]	
**Behavioral/conduct problems**						
Yes	62 (5.2) [3.2–8.3]	<.001	340 (19.9) [16.5–23.8]	<.001	893 (55.8) [50.8–60.7]	<.001
No	172 (1.5) [1.1–2.0]		819 (6.0) [5.4–6.8]		3,577 (25.1) [23.7–26.5]	
**Autism spectrum disorder**						
Yes	19 (4.5) [2.1–9.3]^f^	.01	128 (20.3) [14.9–27.1]	<.001	392 (59.4) [51.0–67.2]	<.001
No	212 (1.7) [1.3–2.2]		1,033 (6.8) [6.1–7.6]		4,083 (26.7) [25.3–28.1]	
**ADHD**						
Yes	56) [3.8) [2.3–6.2]	.002	372 (15.5) [13.0–18.3]	<.001	1,002 (49.2) [44.8–53.6]	<.001
No	173 (1.5) [1.1–2.0]		787 (6.3) [5.6–7.1]		3,449 (25.2) [23.8–26.7]	
**Bullying victimization, past year**						
Never	64 (1.1) [0.7–1.6]	<.001	341 (4.4) [3.7–5.3]	<.001	1,786 (23.3) [21.5–25.3]	<.001
1–2 times in the past year	79 (2.0) [1.2–3.3]		435 (9.4) [8.00–11.0]		1,634 (31.0) [28.8–33.4]	
1–2 times per month	33 (3.0) [1.7–5.2]		196 (14.4) [11.4–18.0]		568 (38.7) [34.2–43.4]	
1–2 times per week	33 (4.5) [2.6–7.5]		107 (15.1) [10.5–21.2]		320 (44.1) [35.9–52.7]	
Almost every day	24 (15.9) [9.4–25.7]		74 (28.7) [19.9–39.4]		144 (54.5) [43.9–64.8]	
**Screentime during weekdays (excluding schoolwork), no. of hours**						
≤1	36 (1.4) [0.8–2.4]	.001	199 (4.6) [3.6–5.9]	<.001	958 (20.1) [17.9–22.5]	<.001
2–3	114 (1.3) [1.00–1.8]		586 (6.3) [5.5–7.2]		2,417 (27.2) [25.5–29.0]	
≥4	81 (3.3) [2.3–4.8]		367 (14.3) [12.0–17.0]		1,070 (41.5) [37.6–45.4]	
**Health care factors**						
**Usual source of sick care**						
Yes	173 (1.5) [1.2–1.9]	.08	902 (7.6) [6.7–8.5]	.50	3,396 (27.7) [26.2–29.3]	.91
No	48 (2.6) [1.5–4.4]		179 (6.9) [5.5–8.7]		772 (28.0) [24.6–31.6]	
**Personal doctor or nurse**						
Yes	170 (1.8) [1.4–2.4]	.98	899 (7.6) [6.8–8.5]	.27	3,472 (27.9) [26.4–29.3]	.77
No	63 (1.8) [1.2–2.8]		256 (6.7) [5.4–8.1]		1,004 (28.4) [25.3–31.7]	
**Preventive medical visit, past year**						
Yes	125 (1.4) [1.0–2.1]	.92	691 (7.0) [6.1–7.9]	.08	2,805 (27.2) [25.6–28.9]	.19
No	27 (1.4) [0.8–2.4]		103 (5.1) [3.7–7.1]		501 (24.3) [20.6–28.4]	
**Doctor ever told caregiver their child is overweight**						
Yes	92 (10.2) [7.4–14.0]	<.001	101 (9.4) [6.9–12.8]	.10	332 (34.1) [29.0–39.7]	.01
No	140 (1.2) [0.8–1.6]		1,056 (7.2) [6.5–8.0]		4,141 (27.5) [26.1–29.0]	
**Family characteristics**						
**Highest household education**						
Less than high school diploma	13 (1.6) [0.7–3.4]^f^	<.001	22 (6.5) [4.0–10.6]	.14	103 (26.4) [19.5–34.7]	.01
High school diploma	45 (3.7) [2.2–6.1]		161 (7.4) [5.7–9.4]		651 (33.2) [29.3–37.3]	
Some college	67 (1.7) [1.2–2.5]		296 (9.2) [7.5–11.3]		1,092 (29.8) [27.1–32.7]	
College degree or higher	110 (1.3) [0.9–1.7]		685 (6.7) [5.9–7.6]		2,646 (25.6) [24.2–27.1]	
**Caregiver mental and emotional health**						
One or both adults excellent/very good	88 (1.2) [0.9–1.8]	.002	478 (5.8) [4.9–6.7]	<.001	2,146 (23.5) [21.8–25.3]	<.001
At least 1 adult good/fair/poor	132 (2.5) [1.9–3.3]		657 (10.2) [9.0–11.6]		2,199 (35.2) [32.9–37.5]	
**Caregiver concern about child’s weight**						
Yes, concerned it’s too high	108 (10.1) [7.4–13.7]	<.001	112 (9.2) [6.8–12.3]	<.001	380 (34.6) [29.4–40.3]	<.001
Yes, concerned it’s too low	29 (6.1) [3.3–11.1]^f^		549 (29.1) [22.1–37.3]		427 (73.5) [66.0–79.9]	
No, not concerned	96 (0.9) [0.6–1.4]		875 (6.3) [5.6–7.1]		3,674 (25.7) [24.3–27.1]	
**Caregiver and child share ideas or talk**						
Very well	125 (1.6) [1.1–2.3]	<.001	547 (5.1) [4.4–5.9]	<.001	2,420 (23.0) [21.4–24.6]	<.001
Somewhat well	79 (1.7) [1.1–2.5]		478 (9.9) [8.5–11.6]		1,674 (34.5) [31.9–37.3]	
Not very well or not well at all	25 (6.5) [3.5–11.5]^f^		125 (23.7) [17.8–30.7]		345 (62.1) [54.6–69.1]	
**Family eats meals together, days per week**						
0	13 (1.9) [0.9–3.9]^f^	.60	60 (15.9) [10.3–23.6]	<.001	180 (43.7) [34.8–53.0]	<.001
1–3	61 (2.1) [1.4–3.2]		336 (10.2) [8.4–12.3]		1,167 (34.7) [31.6–38.1]	
4–6	65 (1.5) [1.0–2.2]		348 (7.5) [6.3–8.9]		1,360 (26.8) [24.7–29.11	
7	90 (1.9) [1.3–2.8]		406 (5.4) [4.6–6.4]		1,718 (24.8) [22.8–27.0]	
**No. of ACEs^g^**						
0	103 (1.1) [0.7–1.6]	<.001	555 (5.0) [4.4–5.8]	<.001	2,677 (23.8) [22.3–25.4]	<.001
1	53 (2.6) [1.6–4.4]		278 (9.8) [8.00–11.9]		912 (32.4) [29.1–35.8]	
≥2	79 (4.4) [3.1–6.1]		331 (14.9) [12.4–17.9]		903 (41.4) [37.2–45.8]	

Abbreviations: ACE, adverse childhood experience; ADHD, attention deficit/hyperactivity disorder; NA, not applicable.

a Overexercising was excluded due to small sample sizes for children aged 6–11 years.

b Restrictive eating behaviors are skipping meals or fasting, purging or vomiting after eating, and/or using diet pills, laxatives, or diuretics.

c Avoidant eating behaviors are low interest in food, extremely picky eating, and/or not eating due to fear of vomiting or choking.

d Determined by Rao–Scott design-adjusted χ^2^ test of independence at significance level of .05.

e Estimate suppressed due to unreliability (relative SE >50%).

f Interpret estimate with caution (relative SE >30%).

g ACEs include parents divorced or separated; parent died; parent incarcerated; interpersonal violence in the home; victim or witness of neighborhood violence; lived with someone who was mentally ill, severely depressed, or suicidal; lived with someone with an alcohol or drug problem; treated unfairly because of race or ethnicity; treated unfairly because of disability or health condition.

**Table 3B:** Caregiver-Reported Prevalence of Disordered Eating–Related Behaviors and Concerns Among Children Aged 6 to 11 Years, by Sociodemographic, Economic, Health-Related, and Family Characteristics, National Survey of Children’s Health, 2022^a^

Characteristic	Binge eating, past year		Caregiver “very much” concerned about their child’s disordered eating behaviors, past year	
	Unweighted no. (weighted %) [95% CI]	*P* value^b^	Unweighted no. (weighted %) [95% CI]	*P* value^b^
**Total**	540 (3.8) [3.3–4.4]	NA	218 (5.2) [4.0–6.6]	NA
**Sociodemographic and economic characteristics**				
**Race and ethnicity**				
Hispanic, Latino, or Spanish origin, any race	103 (4.6) [3.4–6.2]	.25	46 (6.3) [3.9–10.2]	.10
Non-Hispanic American Indian/Alaska Native	—c		—c	
Non-Hispanic Asian	32 (2.0) [1.2–3.3]		10 (5.6) [2.3–12.7]^d^	
Non-Hispanic Black	35 (3.9) [2.6–6.0]		21 (8.4) [4.9–14.2]	
Non-Hispanic Native Hawaiian and other Pacific Islander	—c		—c	
Non-Hispanic White	335 (3.6) [3.0–4.5]		127 (3.7) [2.6–5.2]	
Non-Hispanic multiple races	27 (2.9) [1.6–5.1]		12 (4.0) [1.6–9.8]^d^	
**Household language**				
English	490 (3.9) [3.4–4.6]	.37	189 (5.0) [3.8–6.5]	.75
Spanish	29 (3.6) [2.1–6.2]		16 (6.4) [2.9–13.6]^d^	
Other	18 (2.1) [1.0–4.6]^d^		11 (5.2) [2.2–11.8]^d^	
**Family income-to-poverty ratio, % federal poverty level**				
<100	118 (5.4) [3.8–7.1]	.01	54 (8.9) [4.4–13.3]	.09
100–199	124 (4.3) [2.9–5.8]		36 (4.9) [1.2–8.6]^d^	
200–399	163 (4.0) [2.8–5.1]		63 (4.5) [2.2–6.8]	
≥400	135 (2.4) [1.5–3.3]		65 (3.5) [1.9–5.1]	
**Food insufficiency, past year**				
Always could afford nutritious meals	243 (2.5) [2.0–3.1]	<.001	102 (4.6) [3.2–6.6]	.34
Always could afford enough to eat, but not always nutritious food	227 (6.0) [4.7–7.6]		92 (5.6) [3.8–8.1	
Often or sometimes could not afford enough to eat	60 (9.3) [6.3–13.4]		20 (8.2) [4.0–16.2]^d^	
**Health insurance status**				
Private only	246 (2.6) [2.1–3.4]	<.001	94 (3.2) [2.3–4.4]	<.001
Public (alone or with private)	267 (6.0) [4.9–7.2]		107 (7.7) [5.4–10.8]	
Uninsured	19 (2.5) [1.2–4.9]^d^		11 (3.6) [1.5–8.3]^d^	
**Health status, health-related behaviors, and health care characteristics**				
**General health status**				
Excellent/very good	397 (3.1) [2.6–3.7]	<.001	128 (3.6) [2.6–5.0]	<.001
Good/fair/poor	141 (10.2) [7.7–13.3]		88 (13.4) [9.4–18.8]	
**Body mass index**				
Less than 5th percentile	34 (1.4) [0.8–2.5]	<.001	29 (3.6) [1.9–6.6]^d^	.07
5th to less than 85th percentile	141 (1.5) [1.1–2.0]		98 (4.6) [3.2–6.6]	
85th to less than 95th percentile	100 (5.2) [3.8–7.1]		18 (2.5) [1.1–5.3]^d^	
95th or greater percentile	225 (9.2) [7.5–11.3]		49 (7.4) [4.4–12.4]	
**Current mental, emotional, or behavioral conditions**				
**Depression**				
Yes	69 (21.6) [14.6–30.8]	<.001	23 (11.5) [6.5–19.5]	.006
No	466 (3.4) [2.9–4.0]		193 (4.8) [3.7–6.3]	
**Anxiety problems**				
Yes	171 (11.7) [9.1–14.9]	<.001	83 (9.0) [5.9–13.5]	.004
No	361 (3.0) [2.5–3.6]		130 (4.3) [3.1–5.8]	
**Behavioral or conduct problems**				
Yes	178 (14.5) [11.1–18.7]	<.001	85 (9.7) [6.3–14.7]	.001
No	360 (2.7) [2.3–3.3]		131 (4.2) [3.1–5.6]	
**Autism spectrum disorder**				
Yes	71 (12.9) [8.6–18.8]	<.001	48 (11.2) [6.4–18.8]	.004
No	465 (3.4) [2.9–4.0]		168 (4.6) [3.5–6.0]	
**ADHD**				
Yes	184 (11.1) [8.5–14.3]	<.001	79 (7.4) [4.7–11.6]	.09
No	352 (2.9) [2.5–3.5]		137 (4.7) [3.5–6.2	
**Bullying victimization, past year**				
Never	146 (2.1) [1.6–2.7]	<.001	59 (4.5) [2.8–7.0]	<.001
1–2 times in the past year	188 (4.9) [3.8–6.3]		64 (3.5) [2.3–5.3]	
1–2 times per month	83 (6.9) [5.0–9.4]		39 (5.8) [3.3–10.0]	
1–2 times per week	75 (9.4) [6.3–13.7]		29 (9.2) [4.9–16.7]^d^	
Almost every day	44 (22.2) [14.7–32.3]		103 (18.6) [10.8–30.1]	
**Screentime during weekdays (excluding schoolwork), no. of hours**				
≤1	72 (2.1) [1.2–3.4]	<.001	38 (5.8) [3.2–10.4]^d^	.43
2–3	279 (3.2) [2.7–3.9]		107 (4.4) [3.1–6.2]	
≥4	182 (7.9) [6.2–10.0]		69 (6.3) [4.2–9.4]	
**Health care factors**				
**Usual source of sick care**				
Yes	390 (3.7) [3.1–4.5]	.69	164 (4.8) [3.7–6.2]	.56
No	104 (3.5) [2.6–4.7]		35 (5.8) [3.2–10.3]	
**Personal doctor or nurse**				
Yes	392 (3.7) [3.1–4.4]	.39	156 (4.7) [3.6–6.2]	.38
No	145 (4.2) [3.2–5.5]		58 (6.1) [3.8–9.7]	
**Preventive medical visit, past year**				
Yes	309 (3.7) [3.1–4.5]	.06	122 (4.1) [3.0–5.7]	.34
No	64 (2.4) [1.5–3.7]		11 (2.6) [1.1–6.3]^d^	
**Doctor ever told caregiver their child is overweight**				
Yes	165 (21.6) [17.0–27.1]	<.001	41 (10.1) [6.5–15.4]	.002
No	373 (2.5) [2.1–2.9]		176 (4.5) [3.4–6.0]	
**Family characteristics**				
**Highest household education**				
Less than high school	27 (6.0) [3.7–9.7]	.004	11 (7.8) [3.4–16.9]^d^	.02
High school diploma	115 (5.2) [3.8–7.2]		37 (8.5) [5.0–13.9]	
Some college	138 (3.9) [3.0–5.2]		53 (4.2) [2.8–6.3]	
College degree or higher	260 (2.9) [2.4–3.6]		117 (3.6) [2.7–4.9]	
**Caregiver mental and emotional health**				
One or both adults excellent/very good	177 (2.5) [2.0–3.3]	<.001	90 (5.7) [3.9–8.1]	.31
At least 1 adult good/fair/poor	337 (5.7) [4.8–6.8]		116 (4.4) [3.2–6.0]	
**Caregiver concern about child’s weight**				
Yes, concerned it’s too high	217 (22.9) [28.1–50.9]	<.001	50 (11.0) [7.0–17.0]	<.001
Yes, concerned it’s too low	23 (6.1) [3.1–11.7]^d^		80 (20.0) [13.2–29.0]	
No, not concerned	299 (2.1) [1.8–2.5]		87 (2.6) [1.7–3.9]	
**Caregiver and child share ideas or talk**				
Very well	233 (2.5) [2.0–3.1]	<.001	99 (5.3) [3.7–7.6]	.07
Somewhat well	220 (4.8) [3.8–6.0]		76 (4.0) [2.6–6.01	
Not very well or not well at all	77 (17.3) [12.1–24.0]		37 (9.3) [5.2–15.8]	
**Family eats meals together, days per week**				
0	25 (6.2) [3.6–10.7]	.01	17 (10.3) [4.2–22.9]^d^	.28
1–3	155 (5.7) [4.2–7.5]		60 (4.5) [2.9–7.0]	
4–6	142 (3.3) [2.5–4.5]		55 (4.2) [2.6–6.8]	
7	208 (3.0) [2.5–3.8]		82 (5.7) [3.8–8.5	
**No. of ACEs^e^**				
0	225 (2.3) [1.8–2.8]	<.001	93 (3.4) [2.4–4.8]	.008
1	134 (5.7) [4.1–7.8]		54 (7.5) [4.5–12.3]	
≥2	181 (8.7) [6.9–11.0]		71 (7.4) [5.00–10.9]	

Abbreviations: ACE, adverse childhood experience; ADHD, attention deficit/hyperactivity disorder; NA, not applicable.

a Overexercising was excluded due to small sample sizes for children aged 6–11 years.

b Determined by Rao–Scott design-adjusted χ^2^ test of independence at significance level of .05.

c Estimate suppressed due to unreliability (relative SE >50%).

d Interpret estimate with caution (relative SE >30%).

e ACEs include parents divorced or separated; parent died; parent incarcerated; interpersonal violence in the home; victim or witness of neighborhood violence; lived with someone who was mentally ill, severely depressed, or suicidal; lived with someone with an alcohol or drug problem; treated unfairly because of race or ethnicity; treated unfairly because of disability or health condition.

### Correlates of disordered eating behaviors among adolescents

Given the sex differences among adolescents aged 12 to 17 years, we examined the correlates of disordered eating behaviors and concerns separately for male adolescents (Table 4A and Table 4B) and female adolescents (Table 5A and Table 5B). Patterns of caregiver-reported disordered eating behaviors and related concerns among adolescents were generally similar to those observed among children aged 6 to 11 years. We observed a higher prevalence of several measures among adolescents experiencing food insufficiency and those with public health insurance, compared with food secure and privately insured adolescents, respectively. Non-Hispanic Black male adolescents had higher prevalence of avoidant eating behaviors (27.3%; 95% CI, 22.1%–33.1%) than non-Hispanic White male adolescents (19.8%; 95% CI, 18.3%–21.4%) (Table 4A), and non-Hispanic Asian female adolescents had lower prevalence of avoidant eating behaviors (14.5%; 95% CI, 10.6%–19.6%) than non-Hispanic White females (25.5%; 95% CI, 23.7%–27.3%) (Table 5A). In addition, non-Hispanic multiple race female adolescents had the highest prevalence of restrictive eating behaviors (22.1%; 95% CI, 16.9%–28.2%) and binge eating (12.5%; 95% CI, 8.0%–19.0%) (Table 5B). Caregivers of female adolescents from lower income households were also more likely to report any avoidant eating behaviors (<100% FPL: 31.1%; 95% CI, 25.4%–36.9%), compared with caregivers of female adolescents from higher income households (≥400% FPL: 22.8%; 95% CI, 20.5%–25.0%) (Table 5A).

**Table 4A:** Caregiver-Reported Prevalence of Disordered Eating–Related Behaviors and Concerns Among Male Adolescents Aged 12 to 17 years (N = 9,878), by Sociodemographic, Economic, Health-Related, and Family Characteristics, National Survey of Children’s Health, 2022

Characteristic	Child “very much” concerned about their weight, body shape, or body size, past year		Any restrictive eating behaviors, past year^a^		Any avoidant eating behaviors, past year^b^	
	Unweighted no. (weighted %) [95% CI]	*P* c	Unweighted no. (weighted %) [95% CI]	*P* c	Unweighted no. (weighted %) [95% CI]	*P* c
**Total**	496 (5.0) [4.1–6.0]	NA	1,249 (11.5) [10.3–12.7]	NA	2,197 (20.7) [19.1–22.3]	NA
**Sociodemographic and economic characteristics**						
**Race and ethnicity**						
Hispanic, Latino, or Spanish origin, any race	92 (5.6) [3.8–8.2]	.04	208 (12.8) [10.1–16.2]	.36	340 (19.9) [16.2–24.2]	.008
Non-Hispanic American Indian/Alaska Native	8 (7.3) [3.0–16.8]^d^		9 (6.2) [2.6–14.1]^d^		17 (22.8) [11.3–40.6]^d^	
Non-Hispanic Asian	33 (6.9) [3.9–11.8]		60 (9.9) [6.3–15.1]		76 (12.9) [8.3–19.5]	
Non-Hispanic Black	44 (7.9) [4.3–13.9]		75 (10.1) [6.9–14.7]		196 (27.3) [22.1–33.1]	
Non-Hispanic Native Hawaiian and other Pacific Islander	—e		—e		—e	
Non-Hispanic White	289 (3.9) [3.2–4.6]		782 (10.9) [9.8–12.2]		1,406 (19.8) [18.3–21.4]	
Non-Hispanic multiple races	28 (2.4) [1.4–4.1]		110 (14.3) [10.9–18.5]		159 (22.2) [17.5–27.6]	
**Household language**						
English	440 (4.6) [3.8–5.7]	.17	1,139 (11.1) [10.0–12.3]	.41	2,039 (21.1) [19.5–22.8]	.14
Spanish	32 (5.9) [3.5–9.8]		63 (13.8) [9.4–19.9]		95 (20.4) [14.9–27.2]	
Other	22 (10.1) [3.7–24.6]^d^		41 (14.9) [7.7–27.0]^d^		48 (12.6) [7.7–19.9]	
**Family income-to-poverty ratio, % federal poverty level**						
<100	81 (6.3) [3.3–9.3]	.03	172 (11.0) [7.5–14.5]	.82	328 (22.1) [16.4–27.9]	.74
100–199	101 (5.4) [2.8–8.0]		206 (11.5) [8.4–14.5]		390 (20.3) [16.4–24.2]	
200–399	134 (5.6) [3.6–7.7]		345 (12.4) [9.7–15.1]		618 (21.4) [17.9–25.0]	
≥400	180 (3.3) [2.4–4.2]		526 (10.8) [9.2–12.4]		861 (19.4) [16.9–22.0]	
**Food insufficiency, past year**						
Always could afford nutritious meals	287 (3.9) [3.2–4.8]	.004	766 (9.9) [8.7–11.3]	<.001	1,320 (17.9) [16.3–19.5]	<.001
Always could afford enough to eat, but not always nutritious food	165 (6.7) [4.6–9.8		393 (14.0) [11.5–17.0]		709 (25.0) [21.4–28.9]	
Often or sometimes could not afford enough to eat	36 (9.3) [5.6–15.0]		77 (20.2) [14.5–27.5]		134 (34.6) [27.3–42.7]	
**Health insurance status**						
Private only	275 (3.6) [2.9–4.4]	.03	805 (10.8) [9.5–12.3]	.12	1,367 (19.1) [17.5–20.8]	.28
Public (alone or with private)	192 (7.6) [5.6–10.1]		381 (13.3) [11.1–15.9]		701 (23.0) [20.3–26.0]	
Uninsured	—e		45 (8.5) [4.8–14.6]		80 (19.9) [11.2–33.1]	
**Health status, health-related behaviors, and health care characteristics**						
**General health status**						
Excellent/very good	336 (3.7) [3.0–4.6]	<.001	955 (10.1) [9.0–11.4]	<.001	1,747 (19.0) [17.5–20.7]	<.001
Good/fair/poor	158 (13.5) [9.2–19.4]		292 (20.6) [16.5–25.4]		446 (31.8) [26.6–37.4]	
**Body mass index**						
Less than 5th percentile	26 (6.9) [3.0–15.2]^d^	<.001	115 (14.1) [9.1–21.2]	.22	242 (32.4) [23.5–42.7]	.005
5th to less than 85th percentile	199 (2.5) [2.0–3.2]		712 (10.4) [9.1–11.8]		1,278 (19.5) [17.8–21.4]	
85th to less than 95th percentile	63 (2.8) [2.0–4.1]		162 (13.9) [10.2–18.5]		259 (20.8) [17.2–25.0]	
95th or greater percentile	172 (10.9) [8.1–14.7]		230 (11.4) [9.2–14.0]		375 (19.7) [16.4–23.3]	
**Current mental, emotional, or behavioral conditions**						
**Depression**						
Yes	131 (17.2) [13.2–22.1]	<.001	254 (35.6) [29.8–41.7]	<.001	349 (44.5) [38.4–50.7]	<.001
No	363 (4.3) [3.4–5.4]		984 (10.0) [8.8–11.2]		1,816 (19.0) [17.5–20.7]	
**Anxiety problems**						
Yes	182 (13.2) [9.5–18.0]	<.001	398 (26.0) [22.2–30.3]	<.001	647 (41.0) [36.4–45.8]	<.001
No	306 (3.9) [3.1–4.9]		834 (9.4) [8.2–10.7]		1,515 (18.0) [16.3–19.7]	
**Behavioral or conduct problems**						
Yes	98 (5.6) [4.2–7.6]	.45	257 (21.4) [17.2–26.4]	<.001	454 (38.7) [32.8–45.0]	<.001
No	395 (4.9) [3.9–6.0]		985 (10.4) [9.3–11.7]		1,727 (18.6) [17.0–20.3]	
**Autism spectrum disorder**						
Yes	33 (3.2) [2.0–5.0]	.07	102 (20.5) [14.1–28.9]	.002	266 (48.2) [40.1–56.5]	<.001
No	460 (5.0) [4.1–6.1]		1,137 (11.0) [9.9–12.24]		1,915 (19.3) [17.8–20.9]	
**ADHD**						
Yes	155 (7.3) [5.6–9.5]	.007	412 (19.0) [16.2–22.3]	<.001	692 (31.6) [27.9–35.5]	<.001
No	338 (4.5) [3.5–5.7]		827 (10.0) [8.8–11.4]		1,480 (18.4) [16.7–20.2]	
**Bullying victimization, past year**						
Never	217 (3.5) [2.7–4.5]	<.001	623 (8.9) [7.7–10.4]	<.001	1,128 (17.6) [15.8–19.6]	<.001
1–2 times in the past year	126 (7.9) [5.1–12.0]		335 (15.0) [12.3–18.2]		606 (24.9) [21.8–28.3]	
1–2 times per month	59 (9.1) [6.2–13.2]		147 (22.3) [17.1–28.6]		214 (28.8) [23.4–35.0]	
1–2 times per week	49 (9.6) [6.4–14.0]		81 (26.1) [19.3–34.3]		131 (41.5) [33.2–50.3]	
Almost every day	39 (13.8) [8.8–21.2]		51 (20.6) [13.8–29.6]		89 (44.4) [33.4–56.1]	
**Screentime during weekdays (excluding schoolwork), no. of hours**						
≤1	36 (2.8) [1.6–5.0]	<.001	56 (4.3) [2.8–6.61]	<.001	138 (11.7) [8.7–15.4]	<.001
2–3	189 (3.4) [2.4–4.7]		501 (9.5) [8.0–11.2]		876 (18.1) [15.9–20.6]	
≥4	265 (7.6) [5.9–9.8]		683 (16.4) [14.2–18.7]		1,155 (26.7) [24.1–29.4]	
**Health care factors**						
**Usual source of sick care**						
Yes	368 (5.0) [4.0–6.2]	.28	997 (11.9) [10.6–13.4]	.04	1,649 (20.4) [18.8–22.1]	.82
No	88 (3.9) [2.7–5.7]		162 (9.1) [7.1–11.5]		388 (20.9) [17.2–25.1]	
**Personal doctor or nurse**						
Yes	380 (4.7) [3.8–5.8]	.45	987 (11.3) [10.2–12.6]	.71	1,668 (19.9) [18.4–21.5]	.26
No	114 (5.6) [3.7–8.4]		256 (11.9) [9.3–15.1]		516 (22.2) [18.7–26.1]	
**Preventive medical visit, past year**						
Yes	296 (5.1) [3.9–6.8]	<.001	809 (12.9) [11.3–14.8]	<.001	1,355 (19.7) [17.9–21.6]	.32
No	59 (2.0) [1.4–2.8]		124 (5.4) [4.0–7.3]		322 (17.4) [13.7–21.7]	
**Doctor ever told caregiver their child is overweight**						
Yes	209 (19.2) [14.5–25.0]	<.001	238 (18.9) [14.8–23.8]	<.001	303 (21.4) [17.6–25.7]	.69
No	285 (2.8) [2.2–3.6]		1,007 (10.4) [9.2–11.6]		1,884 (20.5) [18.9–22.2]	
Family characteristics						
**Highest household education**						
Less than high school diploma	8 (1.9) [0.7–5.0]^d^	.01	32 (13.2) [7.9–21.1]	.68	64 (22.9) [15.4–32.7]	.80
High school diploma	99 (7.8) [5.00–11.8]		184 (11.5) [9.2–14.4]		331 (20.0) [16.8–23.6]	
Some college	105 (4.3) [3.1–5.9]		286 (10.2) [8.2–12.6]		538 (19.9) [17.1–23.1]	
College degree or higher	284 (4.7) [3.7–6.1]		747 (11.7) [10.3–13.2]		1,264 (20.8) [19.0–22.8]	
**Caregiver mental and emotional health**						
One or both adults excellent/very good	194 (3.6) [2.6–4.9]	<.001	574 (8.7) [7.5–10.0]	<.001	1,028 (16.0) [14.4–17.8]	<.001
At least 1 adult good/fair/poor	287 (7.4) [5.7–9.5]		655 (16.8) [14.5–19.4]		1,098 (28.2) [25.3–31.4]	
**Caregiver concern about child’s weight**						
Yes, concerned it’s too high	187 (21.0) [15.6–27.5]	<.001	177 (15.1) [11.7–19.1]	<.001	278 (25.3) [20.9–30.4]	<.001
Yes, concerned it’s too low	58 (14.7) [9.3–22.3]		163 (40.2) [31.4–49.8]		261 (67.2) [57.8–75.4]	
No, not concerned	248 (2.4) [1.9–3.2]		908 (9.9) [8.7–11.2]		1,654 (18.2) [16.6–19.9]	
**Caregiver and child share ideas or talk**						
Very well	218 (4.4) [3.1–6.1]	.22	503 (7.6) [6.5–9.0]	<.001	883 (16.6) [14.6–18.9]	<.001
Somewhat well	204 (5.5) [4.3–6.9]		570 (15.3) [13.2–17.8]		984 (23.3) [21.0–25.9]	
Not very well or not well at all	67 (7.0) [4.6–10.4]		164 (22.5) [17.0–29.1]		301 (38.9) [32.1–46.1]	
**Family eats meals together, days per week**						
0	60 (11.1) [5.8–20.5]^d^	.03	144 (26.9) [19.7–35.6]	<.001	214 (37.3) [29.6–45.6]	<.001
1–3	186 (5.3) [4.2–6.8]		456 (14.7) [12.3–17.5]		817 (25.8) [22.7–29.3]	
4–6	117 (4.0) [2.6–6.1]		370 (9.7) [8.2–11.5]		604 (17.0) [14.9–19.3]	
7	126 (4.5) [3.0–6.7]		265 (7.5) [6.1–9.1]		526 (16.1) [13.9–18.6]	
**No. of ACEs^f^**						
0	182 (2.6) [2.1–3.4]	<.001	521 (9.1) [7.8–10.5]	<.001	984 (18.1) [16.1–20.3]	<.001
1	121 (6.2) [4.0–9.6]		288 (10.1) [7.9–12.8]		525 (18.1) [15.6–22.0]	
≥2	193 (9.5) [7.2–12.5]		440 (19.3) [16.3–22.8]		688 (30.5) [26.8–34.5]	

Abbreviations: ACE, adverse childhood experience; ADHD, attention deficit/hyperactivity disorder; NA, not applicable.

a Restrictive eating behaviors are skipping meals or fasting, purging or vomiting after eating, and/or using diet pills, laxatives, or diuretics.

b Avoidant eating behaviors are low interest in food, extremely picky eating, and/or not eating due to fear of vomiting or choking.

c Determined by Rao–Scott design-adjusted χ^2^ test of independence at significance level of .05.

d Interpret estimate with caution (relative SE >30%).

e Estimate suppressed due to unreliability (relative SE >50%).

f ACEs include parents divorced or separated; parent died; parent incarcerated; interpersonal violence in the home; victim or witness of neighborhood violence; lived with someone who was mentally ill, severely depressed, or suicidal; lived with someone with an alcohol or drug problem; treated unfairly because of race or ethnicity; treated unfairly because of disability or health condition.

**Table 4B:** Caregiver-Reported Prevalence of Disordered Eating–Related Behaviors and Concerns Among Male Adolescents Aged 12 to 17 Years (N = 9,878), by Sociodemographic, Economic, Health-Related, and Family Characteristics, National Survey of Children’s Health, 2022

Characteristic	Binge eating, past year		Overexercising, past year		Caregiver “very much” concerned about their child’s disordered eating behaviors, past year	
	Unweighted no. (weighted %) [95% CI]	*P* a	Unweighted no. (weighted %) [95% CI]	*P* a	Unweighted no. (weighted %) [95% CI]	*P* a
**Total**	508 (5.8) [4.8–7.0]	NA	169 (1.7) [1.3–2.3]	NA	135 (4.5) [3.3–6.0]	NA
**Sociodemographic and economic characteristics**						
**Race and ethnicity**						
Hispanic, Latino, or Spanish origin, any race	90 (6.6) [4.3–10.0]	.59	34 (2.6) [1.5–4.5]	.06	33 (6.2) [3.5–10.8]	.28
Non-Hispanic American Indian/Alaska Native	8 (5.6) [2.2–13.56]^b^		—c		—c	
Non-Hispanic Asian	18 (4.7) [2.1–10.4]^b^		11 (1.5) [0.6–3.6]^b^		—c	
Non-Hispanic Black	32 (6.0) [3.2–10.9]^b^		10 (1.0) [0.5–2.1]^b^		11 (3.0) [1.3–6.9]^b^	
Non-Hispanic Native Hawaiian and other Pacific Islander	—c		—c		—c	
Non-Hispanic White	312 (5.1) [4.2–6.3]		89 (1.3) [0.9–1.9		70 (4.0) [2.7–6.0]	
Non-Hispanic multiple races	47 (8.4) [5.2–13.1]		20 (2.4) [1.1–5.3]^b^		15 (5.3) [2.6–10.5]^b^	
**Household language**						
English	468 (5.7) [4.8–6.8]	.04	147 (1.4) [1.1–1.8]	<.001	117 (4.1) [3.0–5.5]	.04
Spanish	24 (3.3) [1.9–5.8]		16 (5.2) [2.6–10.2]^b^		14 (9.4) [4.1–20.4]^b^	
Other	15 (13.5) [5.2–31.0]^b^		—c		—c	
**Family income-to-poverty ratio, % federal poverty level**						
<100	85 (5.6) [3.7–7.5]	.04	27 (2.2) [0.5–3.8]^b^	.86	23 (5.0) [1.6–8.4]^b^	.24
100–199	120 (8.3) [5.3–11.4]		30 (1.5) [0.5–2.5]^b^		30 (6.7) [2.8–10.7]	
200–399	142 (5.9) [3.5–8.3]		38 (1.8) [0.9–2.7]		38 (3.9) [1.5–6.3]^b^	
≥400	161 (4.3) [3.1–5.4]		74 (1.6) [1.0–2.2]		44 (3.1) [1.7–4.6]	
**Food insufficiency, past year**						
Always could afford nutritious meals	252 (4.1) [3.2–5.3]	<.001	106 (1.7) [1.2–2.4]	.13	68 (3.2) [2.3–4.8]	.02
Always could afford enough to eat, but not always nutritious food	196 (8.8) [6.4–12.0]		49 (1.5) [0.9–2.4]		57 (6.7) [4.2–10.5]	
Often or sometimes could not afford enough to eat	51 (10.9) [7.0–16.5]		—c		9 (3.2) [1.5–6.5]^b^	
**Health insurance status**						
Private only	261 (4.7) [3.6–6.1]	.11	106 (1.4) [1.0–2.0]	.46	68 (3.3) [2.2–4.8]	.01
Public (alone or with private)	217 (7.9) [6.1–10.2]		49 (1.8) [1.1–3.0]		61 (6.3) [4.2–9.5]	
Uninsured	—c		—c		—c	
**Health status, health-related behaviors, and health care characteristics**						
**General health status**						
Excellent/very good	329 (4.4) [3.5–5.5]	<.001	149 (1.7) [1.3–2.3]	>.99	75 (3.0) [2.0–4.5]	<.001
Good/fair/poor	177 (15.6) [11.5–20.9]		20 (1.7) [0.8–3.8]^b^		60 (10.2) [6.7–15.3]	
**Body mass index**						
Less than 5th percentile	—c	<.001	—c	.19	12 (2.4) [1.1–5.3]^b^	.002
5th to less than 85th percentile	181 (3.4) [2.6–4.4]		107 (1.6) [1.2–2.2]		59 (3.4) [2.2–5.2]	
85th to less than 95th percentile	72 (6.7) [4.0–11.0]		27 (2.4) [1.2–4.7]^b^		14 (2.3) [1.2–4.5]^b^	
95th or greater percentile	219 (13.3) [10.0–17.3]		25 (1.8) [0.9–3.3]^b^		43 (8.0) [4.7–13.4]	
**Current mental, emotional, or behavioral conditions**						
**Depression**						
Yes	146 (20.2) [15.1–26.6]	<.001	24 (3.8) [2.0–7.0]^b^	.02	50 (12.3) [7.7–19.1]	<.001
No	357 (4.9) [4.0–6.1]		144 (1.6) [1.2–2.2]		85 (3.4) [2.4–5.0]	
**Anxiety problems**						
Yes	208 (15.5) [11.6–20.4]	<.001	41 (2.7) [1.6–4.4]	.08	70 (9.6) [6.5–14.1]	<.001
No	287 (4.4) [3.5–5.6]		126 (1.6) [1.2–2.2]		60 (2.7) [1.7–4.2]	
**Behavioral or conduct problems**						
Yes	164 (14.8) [11.1–19.6]	<.001	22 (1.4) [0.8–2.5]^b^	.49	52 (10.6) [6.8–16.2]	<.001
No	342 (4.9) [3.9–6.1]		146 (1.8) [1.3–2.4]		83 (3.3) [2.2–4.8]	
**Autism spectrum disorder**						
Yes	81 (15.5) [9.9–23.5]	<.001	—c	.06	29 (9.0) [4.8–16.3]^b^	.02
No	424 (5.4) [4.4–6.6]		162 (1.8) [1.3–2.4]		105 (3.9) [2.7–5.4]	
**ADHD**						
Yes	226 (11.9) [9.4–14.9]	<.001	30 (1.5) [0.8–2.7]	.65	61 (5.6) [3.8–8.4]	.21
No	279 (4.6) [3.6–6.0]		138 (1.8) [1.3–2.4]		73 (3.9) [2.6–5.8]	
**Bullying victimization, past year**						
Never	181 (3.8) [2.8–5.1]	<.001	87 (1.5) [1.0–2.2]	.24	53 (3.7) [2.3–5.8]	.08
1–2 times in the past year	143 (7.9) [5.4–11.5]		50 (2.4) [1.6–3.8]		34 (4.4) [2.4–7.8]^b^	
1–2 times per month	80 (15.8) [10.5–23.1]		16 (2.0) [1.0–4.2]^b^		16 (5.4) [2.3–12.3]^b^	
1–2 times per week	43 (13.7) [8.8–20.8]		8 (1.4) [0.7–3.0]^b^		13 (9.9) [4.2–21.7]^b^	
Almost every day	56 (28.5) [19.9–39.1]		—c		16 (11.4) [6.2–20.0]	
**Screentime during weekdays (excluding schoolwork), h**						
≤1	17 (1.8) [0.8–4.1]^b^	.003	21 (1.5) [0.6–3.4]^b^	.78	—c	.29
2–3	183 (5.5) [3.9–7.5]		74 (1.6) [1.0–2.6]		42 (3.2) [1.7–6.0]^b^	
≥4	301 (7.6) [6.1–9.4]		72 (1.9) [1.4–2.8]		85 (5.6) [4.0–7.7]	
**Health care factors**						
**Usual source of sick care**						
Yes	385 (5.7) [4.6–7.1]	.94	123 (1.6) [1.1–2.2]	.35	104 (4.0) [2.9–5.5]	.47
No	91 (5.6) [3.9–8.1]		32 (2.2) [1.2–3.9]		25 (5.3) [2.6–10.4]^b^	
**Personal doctor or nurse**						
Yes	377 (5.7) [4.6–7.0]	.66	127 (1.7) [1.3–2.4]	.93	101 (4.9) [3.5–6.6]	.48
No	128 (6.2) [4.3–9.0]		40 (1.7) [1.0–2.9]		32 (3.7) [1.8–7.3]^b^	
**Preventive medical visit, past year**						
Yes	290 (6.2) [4.8–7.9]	.009	109 (2.0) [1.4–2.8]	.08	79 (3.7) [2.6–5.4]	.68
No	73 (3.3) [2.2–4.9]		18 (1.0) [0.4–2.1]^b^		17 (4.6) [1.9–10.7]^b^	
**Doctor ever told caregiver their child is overweight**						
Yes	193 (17.0) [12.6–22.5]	<.001	41 (4.7) [2.7–8.1]	<.001	43 (6.2) [3.8–10.2]	.09
No	314 (4.2) [3.3–5.2]		127 (1.3) [1.0–1.7]		90 (3.7) [2.6–5.2]	
**Family characteristics**						
**Highest household education**						
Less than high school	17 (6.3) [2.8–13.6]^b^	.68	8 (3.8) [1.6–8.8]^b^	.06	—c	.47
High school diploma	96 (7.1) [4.8–10.2]		22 (1.4) [0.7–2.9]^b^		26 (4.6) [2.6–8.2]	
Some college	133 (5.5) [4.1–7.3]		32 (1.1) [0.7–1.8]		36 (6.2) [3.7–10.3]	
College degree or higher	262 (5.4) [4.1–7.0]		107 (1.7) [1.2–2.3]		68 (3.4) [2.3–5.1]	
**Caregiver mental and emotional health**						
One or both adults excellent/very good	185 (3.4) [2.5–4.56]	<.001	73 (1.4) [0.9–2.2]	.09	48 (4.5) [2.7–7.2]	.83
At least 1 adult good/fair/poor	314 (10.1) [8.0–12.7]		91 (2.3) [1.6–3.3]		86 (4.8) [3.3–6.8]	
**Caregiver concern about child’s weight**						
Yes, concerned it’s too high	222 (21.7) [16.9–27.4]	<.001	16 (2.3) [1.0–5.5]^b^	.45	47 (9.4) [5.9–14.9]	<.001
Yes, concerned it’s too low	26 (6.5) [3.2–12.5]^b^		13 (2.3) [1.0–5.2]^b^		35 (9.7) [5.3–17.0]	
No, not concerned	259 (3.8) [2.9–4.9]		138 (1.5) [1.1–2.1]		53 (2.7) [1.7–4.3]	
**Caregiver and child share ideas or talk**						
Very well	164 (4.1) [2.9–5.8]	<.001	66 (1.4) [0.9–2.3]	.28	39 (2.9) [1.6–5.3]^b^	.04
Somewhat well	248 (7.2) [5.6–9.1]		80 (2.1) [1.4–3.1]		59 (5.3) [3.4–8.1]	
Not very well or not well at all	89 (11.7) [7.7–17.5]		21 (2.3) [1.2–4.2]^b^		36 (7.8) [4.7–12.6]	
**Family eats meals together, days per week**						
0	67 (15.6) [9.1–25.2]	<.001	19 (3.1) [1.7–5.5]	.09	28 (9.3) [5.0–16.6]^b^	.003
1–3	196 (6.5) [4.9–8.5]		70 (2.2) [1.4–3.3]		48 (2.8) [1.8–4.2]	
4–6	114 (4.1) [2.8–5.9]		44 (1.0) [0.7–1.5]		33 (7.1) [4.1–12.2]	
7	124 (5.4) [3.7–7.7]		33 (1.8) [0.9–3.3]^b^		24 (2.8) [1.5–5.3]^b^	
**No. of ACEs^d^**						
0	178 (3.4) [2.6–4.3]	<.001	73 (1.3) [0.8–2.0]	.07	47 (3.3) [1.9–5.5]	<.001
1	120 (5.5) [3.5–8.7]		35 (1.8) [0.9–3.4]^b^		27 (2.2) [1.2–4.2]^b^	
2 or more	210 (12.6) [9.6–16.4]		61 (2.8) [1.9–4.1]		61 (7.9) [5.2–11.7]	

Abbreviations: ACE, adverse childhood experience; ADHD, attention deficit/hyperactivity disorder; NA, not applicable.

a Determined by Rao–Scott design-adjusted χ^2^ test of independence at significance level of .05.

b Interpret estimate with caution (relative SE >30%).

c Estimate suppressed due to unreliability (relative SE >50%).

d ACEs include parents divorced or separated; parent died; parent incarcerated; interpersonal violence in the home; victim or witness of neighborhood violence; lived with someone who was mentally ill, severely depressed, or suicidal; lived with someone with an alcohol or drug problem; treated unfairly because of race or ethnicity; treated unfairly because of disability or health condition.

**Table 5A:** Caregiver-Reported Observed Prevalence of Disordered Eating–Related Behaviors and Concerns among Female Adolescents Aged 12 to 17 Years (N = 9,150), by Sociodemographic, Economic, Health-Related, and Family Characteristics, 2022 National Survey of Children’s Health

Characteristic	Child “very much” concerned about their weight, body shape, or body size, past year		Any restrictive eating behaviors, past year^a^		Any avoidant eating behaviors, past year^b^	
	Unweighted no. (weighted %) [95% CI]	*P* c	Unweighted no. (weighted %) [95% CI]	*P* c	Unweighted no. (weighted %) [95% CI]	*P* c
**Total**	639 (6.3) [5.5–7.2]	NA	1,622 (16.1) [14.8–17.4]	NA	2,532 (26.5) [24.7–28.3]	NA
**Sociodemographic and economic characteristics**						
**Race and ethnicity**						
Hispanic, Latino, or Spanish origin, any race	130 (6.9) [5.2–9.1]	<.001	308 (18.3) [15.2–21.9]	.009	467 (29.7) [25.5–34.4]	.01
Non-Hispanic American Indian/Alaska Native	—d		7 (13.4) [5.6–28.9]^e^		13 (29.7) [16.7–47.1]	
Non-Hispanic Asian	27 (4.4) [2.5–7.9]		86 (14.4) [10.0–20.3]		114 (14.5) [10.6–19.6]	
Non-Hispanic Black	34 (4.4) [2.5–7.8]		91 (12.2) [8.9–16.5]		163 (28.2) [22.6–34.5]	
Non-Hispanic Native Hawaiian and other Pacific Islander	4 (50.0) [16.7–83.4]^e^		—d		7 (46.7) [13.9–82.6]^e^	
Non-Hispanic White	393 (5.9) [5.1–6.9]		994 (15.2) [13.8–16.7]		1,613 (25.5) [23.7–27.3]	
Non-Hispanic multiple races	50 (10.5) [6.9–15.6]		131 (22.1) [16.9–28.2]		155 (24.8) [19.5–31.0]	
**Household language**						
English	574 (6.2) [5.4–7.1]	.07	1,486 (16.1) [14.8–17.5]	.91	2,334 (26.8) [25.1–28.56]	.03
Spanish	45 (8.4) [5.5–12.8]		78 (16.9) [12.0–23.3]		126 (29.7) [21.8–39.0]	
Other	19 (2.9) [1.5–5.6]^e^		50 (15.1) [9.5–23.1]		54 (12.4) [7.8–19.3]	
**Family income to poverty ratio, % FPL**						
<100	95 (6.8) [4.2–9.4]	.017	200 (16.9) [13.1–21.7]	.77	367 (31.1) [25.4–36.9]	.02
100–199	142 (8.9) [6.3–11.5]		282 (16.7) [13.0–20.3]		433 (27.1) [22.4–31.8]	
200–399	172 (5.7) [4.2–7.2]		524 (16.2) [13.8–18.7]		787 (27.2) [23.8–30.7]	
≥400	230 (4.9) [3.9–6.0]		616 (15.1) [13.1–17.0]		945 (22.8) [20.5–25.0]	
**Food insufficiency, past year**						
Always could afford nutritious meals	343 (4.6) [3.8–5.5]	<.001	967 (13.3) [12.0–14.8]	<.001	1,495 (21.3) [19.5–23.3]	<.001
Always could afford enough to eat, but not always nutritious food	223 (8.8) [7.0–11.0]		518 (20.9) [18.0–24.1]		812 (34.1) [30.2–38.2]	
Often or sometimes could not afford enough to eat	61 (13.1) [8.6–19.5]		109 (25.2) [18.0–33.9]		165 (45.0) [35.4–54.2]	
**Health insurance status**						
Private only	368 (5.0) [4.2–5.9]	<.001	1,038 (15.4) [14.0–17.0]	.02	1,553 (23.7) [21.9–25.6]	<.001
Public (alone or with private)	231 (8.8) [7.1–10.9]		495 (18.8) [16.1–21.8]		823 (32.4) [28.7–36.3]	
Uninsured	24 (4.4) [2.3–8.1]^e^		66 (11.0) [6.9–17.1]		101 (22.3) [15.3–31.2]	
**Health status, health-related behaviors, and health care characteristics**						
**General health status**						
Excellent/very good	418 (4.6) [3.9–5.4]	<.001	1,207 (13.9) [12.6–15.3]	<.001	1,913 (23.1) [21.3–25.0]	<.001
Good/fair/poor	221 (16.6) [13.1–20.7]		414 (29.5) [24.5–35.1]		611 (46.5) [40.0–53.2]	
**Body mass index**						
Less than 5th percentile	19 (2.4) [1.2–4.5]^e^	<.001	60 (8.7) [5.6–13.3]	<.001	135 (28.8) [18.4–42.0]	.62
5th to less than 85th percentile	306 (3.8) [3.1–4.7]		1,048 (15.1) [13.6–16.8]		1,707 (26.1) [24.1–28.2]	
85th to less than 95th percentile	121 (10.2) [7.5–13.60]		246 (19.4) [15.7–23.8]		323 (25.1) [20.7–30.1]	
95th or greater percentile	177 (16.0) [12.6–20.1]		239 (21.2) [17.2–25.9]		315 (29.7) [25.0–34.8]	
**Current mental, emotional, or behavioral conditions**						
**Depression**						
Yes	298 (21.3) [17.5–25.7]	<.001	585 (41.0) [35.6–46.56]	<.001	775 (52.6) [46.6–58.5]	<.001
No	339 (4.2) [3.5–5.0]		1,020 (12.5) [11.3–13.9]		1,733 (22.6) [20.8–24.5]	
**Anxiety problems**						
Yes	370 (16.8) [14.2–19.7]	<.001	788 (33.8) [30.3–37.5]	<.001	1,130 (46.7) [43.0–50.4]	<.001
No	256 (3.6) [2.9–4.5]		807 (11.5) [10.2–12.9]		1,354 (21.2) [19.3–23.3]	
**Behavioral or conduct problems**						
Yes	89 (18.6) [13.2–25.6]	<.001	198 (31.4) [24.8–38.8]	<.001	279 (48.9) [40.4–57.4]	<.001
No	547 (5.6) [4.9–6.52]		1,417 (15.3) [14.0–16.7]		2,245 (25.5) [23.7–27.3]	
**Autism spectrum disorder**						
Yes	23 (12.9) [7.0–22.5]^e^	.02	54 (16.4) [10.8–24.2]	.91	112 (50.7) [39.6–61.9]	<.001
No	614 (6.2) [5.4–7.1]		1,562 (16.1) [14.8–17.5]		2,407 (26.1) [24.3–27.9]	
**ADHD**						
Yes	138 (12.9) [9.9–16.6	<.001	337 (26.5) [22.3–31.2]	<.001	495 (43.0) [37.8–48.3]	<.001
No	494 (5.5) [4.7–6.44		1,272 (14.9) [13.6–16.4]		2,021 (24.8) [22.9–26.7]	
**Bullying victimization, past year**						
Never	211 (4.2) [3.4–5.2]	<.001	652 (12.6) [11.0–14.3]	<.001	1,130 (22.4) [20.2–24.8]	<.001
1–2 times in the past year	212 (6.6) [5.2–8.3]		558 (19.4) [16.8–22.2]		835 (31.0) [27.7–34.5]	
1–2 times per month	77 (10.4) [7.0–15.0]		186 (24.9) [19.9–30.7]		263 (34.5) [28.6–40.9]	
1–2 times per week	67 (22.7) [15.3–32.3]		121 (31.1) [22.9–40.8]		157 (45.6) [35.8–55.7]	
Almost every day	65 (31.0) [21.4–42.5]		87 (39.1) [28.7–50.6]		118 (46.8) [36.1–57.8]	
**Screentime during weekdays (excluding schoolwork), h**						
≤1	36 (3.3) [1.9–5.6]	<.001	71 (7.5) [5.00–11.2]	<.001	149 (16.5) [12.1–22.1]	<.001
2–3	240 (4.1) [3.4–5.1]		682 (12.1) [10.6–13.7]		1,116 (23.7) [21.2–26.4]	
≥4	358 (10.5) [8.8–12.5]		852 (24.9) [22.3–27.7]		1,237 (34.6) [31.7–37.6]	
**Health care factors**						
**Usual source of sick care**						
Yes	518 (7.2) [6.3–8.4]	.008	1,301 (18.0) [16.4–19.7]	<.001	1,954 (26.4) [24.6–28.3]	.64
No	83 (4.3) [3.0–6.2]		206 (10.4) [8.2–13.1]		400 (25.2) [20.9–30.1]	
**Personal doctor or nurse**						
Yes	477 (6.3) [5.4–7.3]	.85	1,274 (17.0) [15.6–18.6]	.07	1,968 (27.2) [25.3–29.2]	.27
No	161 (6.4) [4.9–8.4]		342 (14.0) [11.6–16.9]		546 (24.8) [21.2–28.7]	
**Preventive medical visit, past year**						
Yes	377 (6.0) [5.1–7.0]	<.001	1,007 (16.4) [14.8–18.2]	<.001	1,552 (26.8) [24.6–29.2]	<.001
No	62 (2.9) [2.0–4.2]		155 (9.5) [7.2–12.4]		308 (18.1) [15.0–21.7]	
**Doctor ever told caregiver their child is overweight**						
Yes	218 (20.2) [16.1–25.2]	<.001	287 (23.7) [19.3–28.8]	<.001	337 (32.4) [26.6–38.8]	.03
No	416 (4.5) [3.8–5.3]		1,327 (15.2) [13.8–16.6]		2,178 (25.7) [23.9–27.5]	
**Family characteristics**						
**Highest household education**						
Less than high school diploma	30 (8.6) [5.1–14.1]	.09	52 (12.4) [8.3–18.1]	.22	85 (27.4) [19.00–37.8]	.11
High school diploma	107 (7.2) [5.4–9.5]		225 (15.6) [12.6–19.2]		394 (28.0) [24.1–32.2]	
Some college	162 (7.0) [5.4–8.9]		402 (18.3) [15.5–21.5]		663 (30.7) [27.2–34.4]	
College degree or higher	340 (5.1) [4.2–6.1]		943 (16.0) [14.4–17.8]		1,390 (23.8) [21.9–25.9]	
**Caregiver mental and emotional health**						
One or both adults excellent/very good	260 (4.0) [3.3–4.9]	<.001	665 (11.0) [9.7–12.5]	<.001	1,147 (20.1) [18.1–22.1]	<.001
At least 1 adult good/fair/poor	361 (9.9) [8.3–11.8]		906 (24.3) [21.7–27.2]		1,285 (36.7) [33.4–40.1]	
**Caregiver concern about child’s weight**						
Yes, concerned it’s too high	234 (25.3) [20.7–30.5]	<.001	269 (27.4) [22.4–33.0]	<.001	349 (33.2) [28.0–38.9]	<.001
Yes, concerned it’s too low	68 (15.8) [9.6–24.8]		126 (39.1) [25.5–54.6]		168 (61.5) [40.8–78.8]	
No, not concerned	333 (3.5) [2.9–4.3]		1,220 (13.9) [12.6–15.3]		2,000 (24.4) [22.6–26.4]	
**Caregiver and child share ideas or talk**						
Very well	288 (4.9) [4.0–6.0]	<.001	696 (12.5) [11.0–14.1]	<.001	1,136 (21.1) [19.1–23.3]	<.001
Somewhat well	264 (7.2) [5.9–8.8]		711 (19.3) [17.0–21.9]		1,083 (32.0) [28.8–35.4]	
Not very well or not well at all	82 (15.4) [10.4–22.2]		193 (32.3) [25.0–40.6]		280 (48.6) [39.0–58.3]	
**Family eats meals together, days per week**						
0	65 (9.5) [6.2–14.2]	.02	163 (24.0) [17.9–31.2]	<.001	244 (48.3) [38.1–58.7]	<.001
1–3	264 (7.5) [6.1–9.1]		689 (21.6) [18.9–24.5]		991 (31.1) [28.0–34.3]	
4–6	171 (6.1) [4.6–8.0]		484 (15.5) [13.3–17.9]		739 (23.8) [21.2–26.7]	
7	132 (4.8) [3.6–6.4]		262 (9.6) [7.9–11.8]		517 (20.8) [18.0–24.0]	
**Number of ACEs^f^**						
0	222 (3.4) [2.7–4.3]	<.001	614 (11.0) [9.7–12.6]	<.001	1,103 (20.1) [18.2–22.1]	<.001
1	146 (5.9) [4.5–7.9]		401 (15.0) [12.5–17.8]		612 (27.3) [23.2–31.9]	
2 or more	271 (13.7) [11.3–16.6]		607 (29.9) [26.3–33.7]		817 (41.7) [37.7–45.9]	

Abbreviations: ACE, adverse childhood experience; ADHD, attention deficit/hyperactivity disorder; NA, not applicable.

a Restrictive eating behaviors are skipping meals or fasting, purging or vomiting after eating, and/or using diet pills, laxatives, or diuretics.

b Avoidant eating behaviors are low interest in food, extremely picky eating, and/or not eating due to fear of vomiting or choking.

c Determined by Rao–Scott design-adjusted χ^2^ test of independence at significance level of .05.

d Interpret estimate with caution (relative SE >30%).

e Estimate suppressed due to unreliability (relative SE >50%).

f ACEs include parents divorced or separated; parent died; parent incarcerated; interpersonal violence in the home; victim or witness of neighborhood violence; lived with someone who was mentally ill, severely depressed, or suicidal; lived with someone with an alcohol or drug problem; treated unfairly because of race or ethnicity; treated unfairly because of disability or health condition.

**Table 5B:** Caregiver-Reported Observed Prevalence of Disordered Eating–Related Behaviors and Concerns among Female Adolescents Aged 12 to17 Years (N = 9,150), by Sociodemographic, Economic, Health-Related, and Family Characteristics, 2022 National Survey of Children’s Health

Characteristic	Binge eating, past year		Overexercising, past year		Caregiver “very much” concerned about their child’s disordered eating behaviors, past year	
	Unweighted no. (weighted %) [95% CI]	*P* a	Unweighted no. (weighted %) [95% CI]	*P* a	Unweighted no. (weighted %) [95% CI]	*P* a
**Total**	552 (6.0) [5.1–7.0]	NA	146 (1.5) [1.0–2.0]	NA	220 (5.7) [4.6–7.0]	NA
**Sociodemographic and economic characteristics**						
**Race and ethnicity**						
Hispanic, Latino, or Spanish origin, any race	110 (6.6) [4.7–9.2]	.004	29 (2.0) [1.1–3.6]	.54	58 (6.4) [4.3–9.4]	.82
Non-Hispanic American Indian/Alaska Native	—b		—b		—b	
Non-Hispanic Asian	24 (3.9) [2.0–7.3]^c^		9 (0.5) [0.2–1.3]^c^		—b	
Non-Hispanic Black	29 (3.8) [2.0–7.3]^c^		—b		13 (4.2) [1.8–9.2]^c^	
Non-Hispanic Native Hawaiian and other Pacific Islander	—b		—b		—b	
Non-Hispanic White	330 (5.5) [4.6–6.6]		86 (1.2) [0.9–1.7]		118 (5.4) [4.1–6.9]	
Non-Hispanic multiple races	52 (12.5) [8.0–19.0]		15 (1.0) [0.4–2.3]^c^		22 (8.4) [3.9–17.0]^c^	
**Household language**						
English	500 (6.0) [5.1–7.0]	.16	133 (1.5) [1.0–2.1]	.23	190 (5.3) [4.2–6.6]	.42
Spanish	38 (7.5) [4.5–12.5]		10 (2.1) [0.8–5.2]^c^		23 (8.0) [4.4–14.0]	
Other	11 (2.3) [1.0–5.4]^c^		—b		—b	
**Family income-to-poverty ratio, % federal poverty level**						
<100	88 (7.0) [4.3–9.6]	.046	20 (1.6) [0.6–2.6]^c^	.42	32 (6.2) [3.0–9.5]	.72
100–199	111 (8.4) [5.3–11.5]		—		47 (6.6) [3.7–9.4]	
200–399	174 (5.1) [3.7–6.6]		32 (1.0) [0.3–1.7]^c^		74 (4.8) [2.9–6.6]	
≥400	179 (4.8) [3.6–5.9]		76 (1.9) [1.1–2.8]		67 (5.6) [3.2–8.0]	
**Food insufficiency, past year**						
Always could afford nutritious meals	288 (4.4) [3.5–5.4]	<.001	96 (1.4) [0.9–2.1]	.73	123 (5.3) [3.9–7.2]	.66
Always could afford enough to eat, but not always nutritious food	201 (8.0) [6.4–10.0]		39 (1.6) [0.8–3.2]^c^		74 (6.6) [4.7–9.1]	
Often or sometimes could not afford enough to eat	52 (15.2) [9.0–24.4]		8 (2.0) [0.9–4.6]^c^		19 (5.4) [2.5–11.0]^c^	
**Health insurance status**						
Private only	305 (5.4) [4.4–6.7]	.01	102 (1.7) [1.1–2.6]	.03	110 (4.8) [3.5–6.6]	.008
Public [alone or with private]	211 (7.5) [5.8–9.6]		32 (0.7) [0.4–1.2]		99 (7.6) [5.6–10.3]	
Uninsured	25 (3.0) [1.7–5.1]		9 (3.1) [1.2–7.4]^c^		8 (1.7) [0.7–4.5]^c^	
**Health status, health-related behaviors, and health care characteristics**						
**General health status**						
Excellent/very good	336 (3.9) [3.2–4.7]	<.001	116 (1.3) [0.9–2.0]	.17	91 (3.2) [2.3–4.4]	<.001
Good/fair/poor	216 (19.0) [14.8–24.1]		30 (2.2) [1.2–3.8]		129 (13.7) [10.4–17.9]	
**Body mass index**						
Less than 5th percentile	10 (1.5) [0.7–3.4]^c^	<.001	—b	.91	—b	.21
5th to less than 85th percentile	231 (3.6) [2.8–4.7]		112 (1.3) [0.9–1.9]		122 (5.2) [3.9–6.9]	
85th to less than 95th percentile	106 (7.5) [5.4–10.3]		15 (1.2) [0.6–2.7]^c^		27 (3.9) [2.1–7.1]^c^	
95th or greater percentile	193 (18.8) [14.8–23.7]		—b		51 (8.5) [5.8–12.2]	
**Current mental, emotional, or behavioral conditions**						
**Depression**						
Yes	262 (22.1) [17.6–27.4]	<.001	52 (3.7) [2.4–5.9]	<.001	125 (12.3) [9.3–16.2]	.001
No	285 (3.7) [3.00–4.5]		92 (1.1) [0.7–1.8]		91 (3.2) [2.3–4.4]	
**Anxiety problems**						
Yes	319 (15.5) [12.7–18.9]	<.001	74 (1.3) [0.8–2.0]	.10	158 (10.0) [7.8–12.7]	<.001
No	222 (3.5) [2.8–4.5]		68 (2.1) [1.4–3.1]		57 (3.2) [2.2–4.8]	
**Behavioral or conduct problems**						
Yes	116 (27.8) [20.7–36.2]	<.001	—b	NA	51 (12.3) [8.1–18.2]	<.001
No	433 (4.9) [4.1–5.8]		136 (1.4) [1.0–2.0]		167 (5.0) [3.9–6.4]	
**Autism spectrum disorder**						
Yes	36 (15.1) [9.4–23.4]	<.001	—b	NA	21 (12.2) [6.2–22.4]^c^	.02
No	511 (5.8) [4.9–6.8]		143 (1.5) [1.1–2.0]		197 (5.5) [4.4–6.8]	
**ADHD**						
Yes	157 (15.4) [11.8–19.8]	<.001	—b	NA	66 (9.6) [6.6–13.8]	.004
No	389 (4.9) [4.1–6.0]		133 (1.5) [1.1–2.2]		152 (5.0) [3.9–6.4]	
**Bullying victimization, past year**						
Never	178 (3.8) [2.9–5.0]	<.001	68 (1.4) [0.9–2.3]	.002	76 (4.8) [3.4–6.9]	.002
1–2 times in the past year	192 (7.2) [5.7–9.2]		47 (0.8) [0.6–1.2]		71 (4.6) [3.3–6.4]	
1–2 times per month	76 (13.1) [8.0–20.9]		15 (1.7) [0.8–3.7]^c^		30 (9.0) [5.1–15.4]	
1–2 times per week	58 (16.2) [11.1–23.0]		9 (3.8) [1.5–9.3]^c^		27 (14.3) [8.1–24.0]	
Almost every day	44 (26.8) [17.5–38.7]		—b		15 (8.3) [3.5–18.4]^c^	
**Screentime during weekdays (excluding schoolwork), no. of hours**						
≤1	23 (2.2) [1.2–4.3]^c^	<.001	17 (2.8) [1.1–6.7]^c^	.14	11 (3.4) [1.4–8.2]^c^	.002
2–3	196 (4.5) [3.4–6.0]		77 (1.5) [1.0–2.2]		76 (3.8) [2.6–5.5]	
≥4	328 (9.5) [7.9–11.5]		51 (1.0) [0.6–1.7]		132 (7.9) [6.0–10.2]	
**Health care factors**						
**Usual source of sick care**						
Yes	417 (6.3) [5.2–7.5]	.38	121 (1.6) [1.2–2.3]	NA	172 (6.0) [4.7–7.7]	.26
No	99 (5.3) [3.7–7.4]		—b		33 (4.4) [2.6–7.3]	
**Personal doctor or nurse**						
Yes	415 (5.9) [5.0–7.0]	.71	122 (1.5) [1.1–2.1]	.86	174 (6.0) [4.8–7.6]	.46
No	137 (6.3) [4.6–8.6]		23 (1.4) [0.6–3.1]^c^		46 (5.0) [3.1–7.9]	
**Preventive medical visit, past year**						
Yes	307 (6.1) [4.9–7.6]	.016	93 (1.5) [1.0–2.4]	NA	114 (5.5) [4.1–7.4]	.003
No	68 (3.6) [2.4–5.3]		—b		13 (1.8) [0.9–3.7]^c^	
**Doctor ever told caregiver their child is overweight**						
Yes	208 (16.5) [13.0–20.6]	<.001	17 (1.8) [0.7–4.5]^c^	.67	63 (10.2) [7.0–14.7]	.001
No	341 (4.6) [3.8–5.7]		128 (1.4) [1.0–2.0]		155 (4.9) [3.8–6.3]	
**Family characteristics**						
**Highest household education**						
Less than high school diploma	27 (5.7) [3.0–10.4]^c^	.63	—b	.19	12 (6.4) [3.0–13.2]^c^	.89
High school diploma	99 (7.0) [4.8–10.1]		10 (0.7) [0.3–1.8]^c^		33 (4.9) [3.0–7.9]	
Some college	153 (6.5) [5.00–8.5]		27 (1.2) [0.6–2.2]^c^		54 (5.4) [3.6–8.0]	
College degree or higher	273 (5.4) [4.3–6.7]		102 (1.9) [1.2–3.0]		121 (6.0) [4.4–8.2]	
**Caregiver mental and emotional health**						
One or both adults excellent/very good	199 (3.8) [2.9–5.0]	<.001	77 (1.1) [0.6–1.8]	.03	79 (4.4) [3.0–6.4]	.07
At least 1 adult good/fair/poor	333 (9.4) [7.8–11.4]		65 (2.2) [1.5–3.3]		133 (6.7) [5.1–8.8]	
**Caregiver concern about child’s weight**						
Yes, concerned it’s too high	234 (24.9) [20.0–30.6]	<.001	—b	<.001	57 (9.3) [6.5–13.3]	<.001
Yes, concerned it’s too low	27 (15.9) [7.5–30.6]^c^		25 (7.8) [3.8–15.3]^c^		63 (29.4) [19.3–42.0]	
No, not concerned	288 (3.3) [2.6–4.0]		106 (1.2) [0.8–1.7]		99 (3.2) [2.3–4.4]	
**Caregiver and child share ideas or talk**						
Very well	192 (3.6) [2.7–4.8]	<.001	65 (1.3) [0.8–2.1]	.008	75 (3.9) [2.7–5.6]	.004
Somewhat well	270 (8.6) [7.0–10.5]		63 (1.3) [0.9–1.9]		104 (6.2) [4.5–8.4]	
Not very well or not well at all	84 (15.3) [10.2–22.4]		16 (4.5) [1.9–10.1]^c^		39 (10.8) [6.4–17.4]	
**Family eats meals together, days per week**						
0	61 (9.4) [6.3–14.0]	.006	11 (1.0) [0.5–2.2]^c^	.64	34 (8.8) [4.9–15.4]	.19
1–3	216 (7.7) [5.9–10.0]		58 (1.7) [1.1–2.6]		87 (5.9) [4.2–8.2]	
4–6	159 (5.5) [4.0–7.4]		48 (1.2) [0.6–2.1]^c^		49 (3.9) [2.6–6.0]	
7	109 (4.3) [3.2–5.8]		28 (1.6) [0.8–3.3]^c^		49 (6.2) [3.9–9.7]	
**No. of ACEs^d^**						
0	173 (3.5) [2.6–4.6]	<.001	78 (1.3) [0.8–2.3]	.07	77 (4.9) [3.3–7.1]	.02
1	127 (5.8) [4.1–8.2]		25 (0.9) [0.5–1.7]^c^		43 (3.9) [2.5–6.0]	
≥2	252 (12.5) [10.1–15.3]		43 (2.4) [1.5–3.9]		100 (8.0) [5.9–10.8]	

Abbreviations: ACE, adverse childhood experience; ADHD, attention deficit/hyperactivity disorder; NA, not applicable.

a Determined by Rao–Scott design-adjusted χ^2^ test of independence at significance level of .05.

b Estimate suppressed due to unreliability (relative SE >50%).

c Interpret estimate with caution (relative SE >30%).

d ACEs include parents divorced or separated; parent died; parent incarcerated; interpersonal violence in the home; victim or witness of neighborhood violence; lived with someone who was mentally ill, severely depressed, or suicidal; lived with someone with an alcohol or drug problem; treated unfairly because of race or ethnicity; treated unfairly because of disability or health condition.

In terms of health-related factors, both male and female adolescents in good/fair/poor general health, those with current mental, emotional, or behavioral conditions, those experiencing more frequent bullying victimization, those with higher levels of screentime, and those whose doctor had ever told their caregivers that they were overweight were observed to have a higher prevalence of most outcomes compared with their counterparts who did not experience these factors. Adolescents of both sexes with higher BMI percentile were also more likely than those with lower BMI percentile to have caregivers report binge eating and child’s concerns about their weight, shape, or size.

In terms of family factors, both male and female adolescents with caregivers in good/fair/poor mental/emotional health, adolescents whose caregivers were concerned about their child’s weight, adolescents whose caregivers did not share ideas or talk together, adolescents from families that never or rarely eat meals together, and adolescents with 2 or more adverse childhood experiences were observed to have a higher prevalence of most caregiver-reported outcomes compared with their counterparts.

Patterns of association among adolescents differed from those among children in 2 notable ways. First, factors related to health care access (ie, preventive medical visits, usual source of sick care), while not significantly associated with disordered eating behaviors and concerns among children, emerged as significant among adolescents; we observed a higher prevalence of behaviors and related concerns among adolescents whose caregivers reported greater access to health care compared with those with less reported access to health care. For example, adolescent females with a preventive visit in the past year were found to have a higher prevalence of restrictive eating behaviors (16.4%; 95% CI, 14.8–18.2), relative to those with no past-year preventive visit (9.5%; 95% CI, 7.2–12.4) (Table 5A). Second, while household education level was significantly associated with disordered eating behaviors and related caregiver concerns among children, we found no significant associations by household education level among either female or male adolescents.

## Discussion

Eating disorders often begin in childhood or adolescence, have among the highest case fatality rate of any mental health condition, and present substantial social and economic costs (2,20). Ongoing national surveillance is needed to provide up-to-date epidemiologic data on these outcomes and track trends over time. This study found a concerningly high prevalence of caregiver-reported eating disorder–related behaviors, especially among adolescents aged 12 to 17; one-third of adolescents were reported by their caregivers to be “somewhat” or “very much” concerned about their body weight, shape, or size and nearly one-third were reported to have engaged in at least 1 form of disordered eating, with even higher levels observed among female adolescents for select restrictive-type and avoidant-type behaviors. These estimates are consistent with extant studies of self-reported disordered eating among young people (6,8), which have documented prevalences of approximately 20%. They are also consistent with the wealth of studies documenting sex-related differences in both disordered eating and body image concerns, which are likely driven by a complex interplay of biologic factors (eg, pubertal onset) and social experiences (eg, intensified appearance pressures) that often characterize adolescence yet disproportionately elevate risk of restrictive-type disordered eating for girls (21).

Prevalence of disordered eating was not negligible among children aged 6 to 11 years either, especially for avoidant-type behaviors (eg, 24% were reported to have engaged in extremely picky eating). Emerging research on disordered eating and body dissatisfaction among children suggests that avoidant-type behaviors are increasingly common among children younger than 12 years and that early signs of body dissatisfaction and related disordered eating may emerge during these years (12,22,23). We did not find evidence of any sex-related eating behavior differences among children, unlike among adolescents, possibly because such differences do not emerge until adolescence (21). Conversely, sex-related differences among adolescents may be an artifact of caregivers being less likely to recognize disordered eating and/or body image concerns among male adolescents due to stigma and stereotypes.

We found significant associations between sociodemographic and economic characteristics and concerns about body weight/shape/size and disordered eating. Specifically, children and adolescents belonging to some racial and ethnic minority groups and those experiencing food insufficiency, who were publicly insured, and were from lower income households had higher prevalences of most outcomes compared with their more advantaged peers, with the largest gaps observed among female adolescents. These results extend a burgeoning literature that challenges prevailing notions about who is affected by eating disorders and related outcomes, by revealing differential risks according to racial and ethnic group and socioeconomic status, likely due to varying sociocultural experiences and exposures (8,14,24).

We found that caregiver-reported body image concerns and disordered eating behaviors among children and adolescents were strongly associated with a range of mental, emotional, and behavioral conditions, as well as with factors related to health care access. The associations between mental, emotional, and behavioral conditions and disordered eating-related outcomes are consistent with prior research documenting high rates of psychiatric comorbidities among adolescents with eating disorders (25); we extend this work by showing that such comorbidities may be present among younger children as well. Our finding that greater health care access was positively associated with concerns about body weight/shape/size and disordered eating among adolescents was somewhat surprising; we hypothesize that caregivers who regularly interact with the health care system on behalf of their child may have more awareness about child and adolescent health, and thus may be more likely to detect and report their child’s body concerns and/or disordered eating. More research is needed to explore this hypothesis. We also note that prevalence of disordered eating behaviors and concerns was higher among children and adolescents whose doctor told their caregiver that they were overweight, and that differences in prevalence by weight status emerged in childhood and widened in adolescence, findings which are consistent with and extend the literature that identifies weight stigma as a key driver of body dissatisfaction and disordered eating among young people (26).

This study has several limitations. First, while the caregiver perspective is valuable, it may underestimate the prevalence of disordered eating behaviors, particularly for those that occur in secrecy or those that their children do not disclose. Additionally, caregivers may be less likely to recognize or report behaviors that do not align with their beliefs or perceptions about disordered eating, especially for behaviors that may initially present as less severe, potentially underestimating the magnitude of the problem for some population groups. Another limitation is the small sample sizes for some behaviors and subgroups, which produced unreliable estimates that should be interpreted with caution. The potential for misclassification also exists, particularly if caregivers interpreted some survey questions as referring to milder eating behaviors, such as picky eating, rather than more serious disordered eating patterns. Finally, the study’s cross-sectional design precludes causal inferences; while we identified numerous associations between disordered eating patterns and various factors, we cannot confirm the temporal ordering of these relationships.

Despite these limitations, this study has several strengths. It provides up-to-date, nationally representative estimates of caregiver-reported disordered eating behaviors and concerns among US children and adolescents, and the use of an annual survey will allow for tracking changes in these measures over time. The inclusion of children aged 6 to 11 years is another strength because it broadens the scope of the existing literature to include age groups that are often overlooked in research on disordered eating. Furthermore, the study captures the caregiver perspective, which is crucial to consider for promoting access to prevention, screening, diagnosis, and treatment for children and adolescents.

Addressing eating disorders among young people at a national level has important implications. From a research perspective, future studies could compare caregiver-reported data with data reported by children and adolescents, examine how caregiver perceptions affect access to care for disordered eating, explore the drivers behind observed differences between groups, and investigate potential interactions between various sociodemographic and economic factors and eating disorders. The results also suggest a need for ongoing population-based data collection on disordered eating behaviors among young people to inform efforts to reduce eating disorders and improve mental health (27). The findings underscore the need for diagnosis, treatment, and resource allocation, particularly for young populations who may be disproportionately affected (28–30). Prevention efforts (eg, active monitoring, caregiver interviews), could be helpful in identifying and addressing eating disorders before they become more severe; more research is needed to develop and test pediatric screening tools and to assess the balance of benefits and harms of universal screening (31,32). Also needed are increased awareness and education for caregivers to recognize and address disordered eating behaviors early as well as for health care providers to identify eating disorders among children and adolescents and engage families in prevention and treatment (33,34).
